# Advancements in nanomedicine for the therapeutic regulation of efferocytosis: opportunities and challenges

**DOI:** 10.7150/thno.128155

**Published:** 2026-03-25

**Authors:** Zehuan Lin, Xiyue Zhou, Shuyun Liu, Meihua Wan, Jingping Liu

**Affiliations:** 1 Department of General Surgery and NHC Key Laboratory of Transplant Engineering and Immunology, Frontiers Science Center for Disease-related Molecular Network, West China Hospital, Sichuan University, Chengdu 610041, China.; 2 West China Center of Excellence for Pancreatitis, Institute of Integrated Traditional Chinese and Western Medicine, West China Hospital, Sichuan University, Chengdu 610041, China.

**Keywords:** efferocytosis, nanomedicine, inflammation, metabolism, target drug delivery, macrophage

## Abstract

Efferocytosis is a conserved event that plays an essential role in maintaining tissue homeostasis and immune balance. However, dysregulation of efferocytosis can induce disturbed apoptotic cells clearance and immune disorders, fueling an array of diseases, such as inflammatory diseases, autoimmunity, and cancers. The process of efferocytosis is dynamic and regulated by a complicated network composed of intracellular pathways and intercellular interactions, which pose significant challenges to current therapies. In recent years, nanomedicine-based strategies have shown potential in the precise regulation of efferocytosis for preventing or reversing pathology in diverse diseases. Although these results are encouraging, the opportunities and challenges in this field remain elusive and need to be comprehensively reviewed, which will be helpful for its improvement and future clinical translation. Here, we briefly introduce the key steps and signaling pathways of efferocytosis and its clinical relevance in diverse diseases. We highlight current advancements in nanomedicines for modulating efferocytosis, especially their design principles and therapeutic applications across a broad range of diseases, such as chronic inflammation, cardiovascular diseases, autoimmune diseases, neurodegenerative diseases and cancers. We discuss the ongoing clinical trials and discuss the challenges and limitations in this field, which may provide insights into developing precision nanomedicines to modulate efferocytosis for treating diverse forms of diseases.

## Introduction

Efferocytosis, a process by which phagocytes recognize and engulf apoptotic cells, is fundamental to maintain tissue homeostasis and the resolution of inflammation. Every day, billions of cells undergo apoptosis and must be cleared efficiently to prevent secondary necrosis and aberrant immune activation 1. The process of efferocytosis is orchestrated by a cascade of molecular “find me” signals released by apoptotic cells, “eat me” signals displayed on their surface, and subsequent receptor recognition and digestion by phagocytes, which ensure that apoptotic debris is removed in an immunologically silent manner, along with the release of various immunoregulatory mediators (e.g., IL-10, TGF-β and lipid mediators) that promote inflammation resolution post-injury. Conversely, defective efferocytosis can result in the accumulation of apoptotic cells and cellular debris, fueling chronic inflammation and autoimmunity 2. Interestingly, efferocytosis can play distinct roles in different conditions. For example, insufficient efferocytosis can exacerbate diseases such as atherosclerosis, autoimmune disorders and neurodegeneration, while tumor-associated macrophage (TAM)-mediated efferocytosis can contribute to the immunosuppressive tumor microenvironment 3-6. Nevertheless, increasing evidence has indicated that precise modulation of efferocytosis may offer a promising therapeutic avenue for treating a variety of diseases.

Currently, some pharmacological approaches (e.g., small molecules, proteins) have been developed to manipulate efferocytosis, but their therapeutic potency is still limited for several reasons. For example, systemic blockade of the “don't-eat-me” signal CD47 with antibodies elicits macrophages to engulf tumor or plaque cells, but it can cause severe side effects such as anemia due to indiscriminate clearance of red blood cells 7. Small-molecule modulators of efferocytic pathways (e.g., MerTK tyrosine kinase inhibitors) show promise in preclinical models, but their short *in vivo* half-time, poor bioavailability and lack of tissue or cell specificity often cause off-target effects and adverse reactions 8. In recent years, nanomedicines have emerged as a robust means for the precise regulation of efferocytosis. For instance, various nanocarriers that can target efferocytic effector cells in distinct conditions (e.g., proinflammatory macrophages in plaques, TAMs in tumors) have been reported 9, 10, which can specifically modulate efferocytosis via either delivering diverse types of payloads (e.g., pathway inhibitors or agonists) 11 or mimicking the surface signals (e.g., the “eat me” signal phosphatidylserine) of apoptotic cells 12. These advanced nanomedicines might have the potential to overcome the drawbacks of conventional treatments, enabling precise regulation of efferocytosis in diverse disease settings. Although the existing findings are encouraging, some critical problems, such as proper selection of targets, design and engineering strategies of nanomedicine, their mechanisms of action, and possible limitations, remain elusive and need to be comprehensively reviewed, which will be helpful for improvement and future clinical translation of these therapies.

In this review, we briefly introduce the key steps and signaling pathways of efferocytosis and its clinical relevance in diverse disease conditions. We highlight the advancements in nanomedicines for modulating efferocytosis, particularly in their design principles and therapeutic applications across a broad range of diseases, such as cancers, cardiovascular diseases, autoimmune and inflammatory disorders and neurodegenerative diseases. We also introduce the ongoing clinical trials and discuss the challenges and limitations in this field, which may provide insights into developing precision nanomedicines to modulate efferocytosis for treating diverse forms of diseases.

## 1. Biology of efferocytosis and its clinical relevance

Efferocytosis, a programmed process of apoptotic cells clearance by phagocytes, plays an essential role in regulating tissue homeostasis and immune reactions. In recent years, a growing body of studies has markedly expanded our understanding of this evolutionarily conserved process, revealing its central role in a broad spectrum of physiological and pathological contexts. These investigations have underscored the importance of efferocytosis in modulating innate immune responses, while disturbed efferocytosis may act as a key pathogenic driver in diverse diseases, such as chronic inflammatory disorders, autoimmune diseases, tumor immune evasion, and neurodegenerative conditions 2, 3, 5. This section provides a concise overview of the key steps and pathways of efferocytosis and discusses the role of efferocytic dysregulation in the pathology of various diseases.

### 1.1. Key stages and signaling pathways of efferocytosis

Briefly, homeostatic efferocytosis comprises several key continual and coordinated steps, including recruitment, recognition, internalization, and degradation 13. The process is initiated by the release of chemoattractants (“find-me” signals) from apoptotic cells, which serve to recruit phagocytes. Subsequent recognition is mediated by specific ligand-receptor interactions (“eat-me” signals). Cytoskeletal rearrangement of phagocytes facilitates the internalization of apoptotic cells, followed by phagosome-lysosome fusion and subsequent degradation of the cellular contents. These sequential events are tightly regulated by a network of molecular pathways that integrate external and intracellular signals to ensure efficient clearance, modulate immune cell trafficking, and promote anti-inflammatory responses.

**Figure 1:**
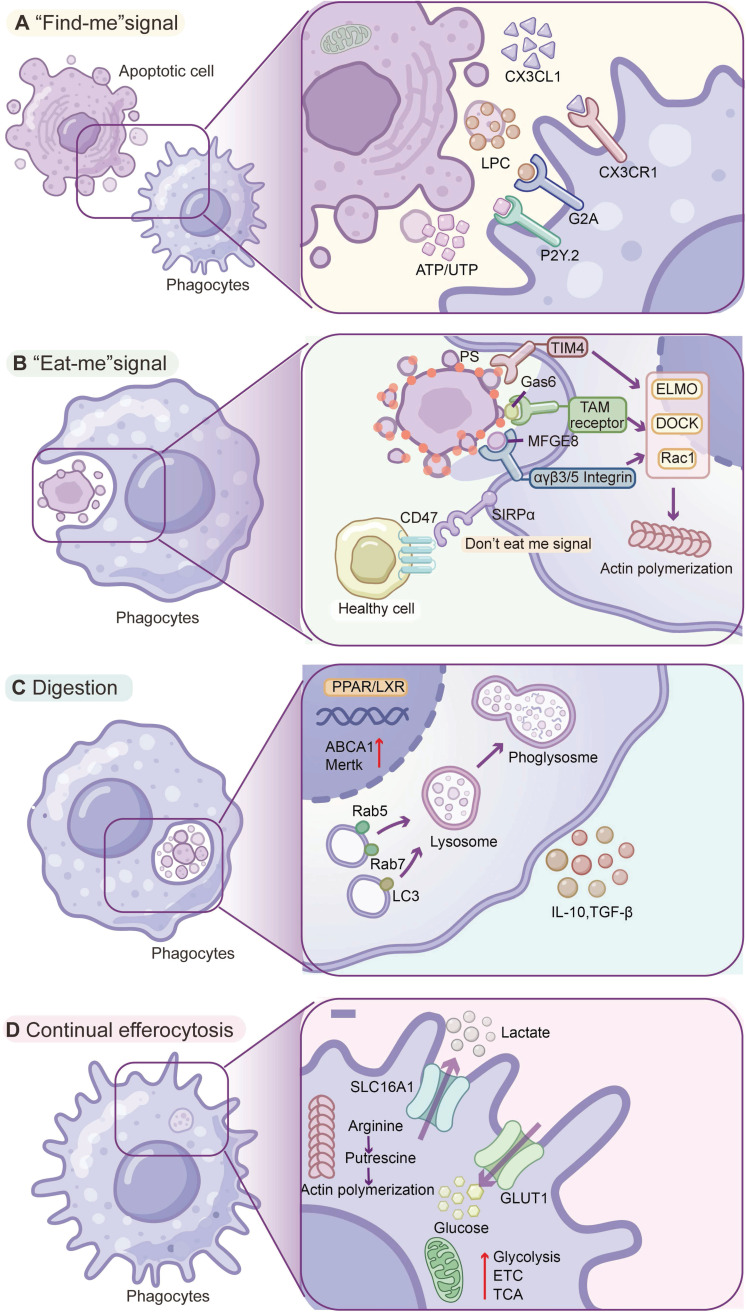
** Key stages and regulatory circuits of efferocytosis. (A) Find-me cues and recruitment:** Apoptotic cells release ATP/UTP, LPC, and CX3CL1, which are sensed by P2Y2, G2A, and CX3CR1 on phagocytes, respectively, establishing chemotactic gradients that attract macrophages/dendritic cells. **(B) Eat-me versus don't-eat-me recognition:** Externalized phosphatidylserine (PtdSer) on apoptotic cells is bridged by MFG-E8 and Gas6 to αvβ3/β5 integrins and the TAM receptor (Tyro3, Axl, MerTK), or bound directly by TIM-4, initiating uptake; healthy cells are protected by the inhibitory CD47-SIRPα checkpoint. Downstream, the ELMO-DOCK complex activates Rac1 to drive actin remodeling, phagocytic cup formation, and internalization. **(C) Digestion and resolution:** Efferosomes mature and fuse with lysosomes (Rab5-to-Rab7 transition with LAP), leading to cargo degradation and release of IL-10 and TGF-β; LXR/PPAR programs upregulate ABCA1 for cholesterol efflux and maintain MerTK expression, supporting receptor availability for subsequent rounds. **(D) Continual efferocytosis and metabolic coupling:** Multiple rounds of uptake require metabolic rewiring—GLUT1-dependent glucose influx and a transient glycolytic burst with SLC16A1-mediated lactate export facilitate receptor recycling and proresolving signaling; mitochondrial FAO and the ETC sustain energy demands; apoptotic-cargo-derived arginine is converted via ARG1/ODC1 to putrescine, stabilizing Rac1 signaling and actin dynamics to maintain multiround efferocytosis. **Abbreviations:** AC: apoptotic cell; PtdSer: phosphatidylserine; TAM: Tyro3/Axl/MerTK family; LAP: LC3-associated phagocytosis; FAO: fatty-acid β-oxidation; ETC: electron transport chain.

In the “find me” stage, apoptotic cells release soluble chemoattractant signals, such as nucleotides (e.g., ATP, UTP), lipids (e.g., lysophosphatidylcholine, LPC), sphingosine-1-phosphate (S1P), and chemokines (e.g., CX3CL1), that serve as “find me” signals to create a gradient to recruit phagocytes (e.g., macrophages, dendritic cells) to the vicinity. These factors are likely actively released by apoptotic cells in a caspase-dependent manner before the cell membrane loses integrity and further bind to corresponding cognate receptors of phagocytes. For example, extracellular ATP/UTP, CX3CL1, and LPC can be detected by P2Y2 receptors, CX3CR1, and G2A receptors of phagocytes, respectively 14. These chemoattractant-receptor interactions ensure that phagocytes are efficiently recruited to sites of cell death (**Figure 1A**).

In the “eat me” stage, phagocytes recognize and bind apoptotic cells via specific surface cues. One of the most universal “eat me” signals is the externalization of phosphatidylserine (PtdSer) on the outer leaflet of the apoptotic cell membrane 15. Phagocytes can bind PtdSer indirectly by bridging molecules (opsonins) or dedicated receptors. Key opsonins, including milk fat globule EGF-factor 8 (MFG-E8) and growth arrest-specific 6 (Gas6), bind PtdSer on apoptotic cells and simultaneously engage phagocyte receptors 13 MFG-E8 bridges PtdSer to integrins (α_v_β_3_/β_5_) of phagocytes, and Gas6 (or Protein S) links PtdSer to the TAM family receptor tyrosine kinases (Tyro3, Axl, MerTK) of phagocytes 16, 17. Some receptors on phagocytes can directly bind apoptotic cells. TIM family receptors (e.g., TIM-4) possess immunoglobulin domains that bind PtdSer 18 (**Figure 1B**); brain-specific angiogenesis inhibitor 1 (BAI1) can also directly bind PtdSer and initiate internalization via the ELMO/Dock/Rac signaling pathway 19.

Crucially, healthy living cells can avoid being mistakenly engulfed by displaying “don't eat me” signals 20 (**Figure 1B**). The prototypical don't-eat-me signal is CD47, a cell-surface protein ubiquitously expressed on host cells, which engages the receptor SIRPα on macrophages 21. When SIRPα binds CD47, it delivers an inhibitory signal (via SHP-1/SHP-2 phosphatases) that halts the phagocytic machinery 21. Recent studies have also identified CD24 on viable cells, which binds Siglec-10 on macrophages as a don't-eat-me signal, particularly exploited by cancer cells with high CD24 expression to suppress phagocytosis 21. Through the balance of “eat me” signals versus “don't eat me” signals, phagocytes are able to discriminate target cells, thereby maintaining self-tolerance while clearing apoptotic cells.

Upon recognition and binding of apoptotic cells, phagocytes initiate engulfment by extending their plasma membrane around target cells. This process relies on phagocyte cytoskeletal rearrangement driven by actin polymerization 22. In phagocytes, the transduction of signals from the engaged receptors converges on small GTPases (e.g., Rac1, Cdc42, and RhoA) that regulate actin, and activation of Rac1 and Cdc42 is required to drive the formation of phagocytic cups and then internalization of apoptotic cells 23 (**Figure 1C**). The TAM receptor MerTK or integrin engagement can trigger intracellular engulfment pathways. MerTK activation leads to recruitment of an ELMO1-Dock180 complex that acts as a guanine nucleotide exchange factor (GEF) to activate Rac1, inducing actin polymerization and membrane zippering around the corpse 24. The dynamic cytoskeletal dance ensures that apoptotic cells can be fully enclosed in a membrane-bound phagosome (termed the efferosome when containing apoptotic cargo) within the macrophage.

Finally, in the digestion stage, after the phagocyte membrane encloses an apoptotic cell, the formed efferosome undergoes a series of maturation processes, including Rab5-to-Rab7 transition, phosphatidylinositol-3-phosphate (PI3P) acquisition, progressive acidification, and fusion with lysosomes for cargo degradation 1. This process can be regulated by a noncanonical autophagy pathway, LC3-associated phagocytosis (LAP), in which autophagy-related proteins (e.g., LC3-II conjugation machinery and Beclin-1/Vps34 complexes) are recruited to single-membrane phagosomes rather than double-membrane autophagosomes 25 (**Figure 1C**). LAP leads to LC3 lipidation on the efferosome and thus promotes phagosome fusion and cargo digestion, which also actively suppresses proinflammatory signaling during apoptotic cells processing 23. In brief, efferocytosis is a highly orchestrated process encompassing the release of find-me signals, specific recognition of apoptotic signals, engagement of phagocytic receptors, cytoskeleton-mediated engulfment, and LC3-assisted phagolysosomal digestion. This event culminates in the secretion of pro-decomposition mediators and the suppression of inflammation, thereby facilitating tissue repair and homeostasis.

### 1.2. Role of metabolic regulation in continual efferocytosis

An important feature of efferocytosis is the ability of phagocytes to ingest multiple apoptotic cells (ACs) over a period, referred to as continual efferocytosis. Continual efferocytosis can ensure efficient clearance when the number of ACs markedly exceeds that of macrophages. However, the massive materials (e.g., lipids, proteins, and nucleotides) from the engulfed ACs can induce metabolic overloading in phagocytes, and thus phagocytes must adapt metabolic rewiring to maintain continual efferocytosis. Upon engulfment of ACs, phagocytes rapidly upregulate glycolysis to meet the bioenergetic and redox demands of phagocytosis 26, and this transient “glycolytic burst” replenishes NAD⁺ (via conversion of pyruvate to lactate) and generates ATP to support actin-dependent engulfment (**Figure 1D**). Mitochondria have emerged as key regulators of continual efferocytosis. Mitochondrial oxidative metabolism plays a dominant role following the initial glycolytic burst, in which fatty acid β-oxidation supplies acetyl-CoA to the tricarboxylic acid (TCA) cycle and sustains electron transport chain (ETC) activity to fuel continual efferocytosis 27. ACs-derived lipids can activate LXR and PPAR nuclear receptors, which also orchestrate a transcriptional program coupling metabolic shift with enhanced efferocytic capacity 28. LXR/PPAR signaling upregulates genes for cholesterol efflux (e.g., ABCA1) to prevent lipid overload and drive the expression of MerTK and opsonins, thereby sustaining phagocytic receptor availability and function 28.

Other metabolic pathways are also involved in regulating continual efferocytosis. For example, arginine catabolism from the apoptotic cargo can be repurposed to sustain continual efferocytosis. ACs-derived arginine is metabolized by arginase 1 (ARG1) to ornithine and then by ornithine decarboxylase 1 (ODC1) to putrescine, and these polyamines can stabilize Dbl (MCF2) mRNA via HuR and thus activate Rac1 to drive actin remodeling and sustain continual efferocytosis 29. Collectively, the current findings indicate that metabolic regulation of phagocytes is essential for sustaining, while disruption of this fine-tuned process can contribute to the onset and progression of diverse pathological conditions.

### 1.3. Pathological role of dysregulated efferocytosis in diverse diseases

Notably, defective or dysregulated efferocytosis has been recognized as a key pathological factor in a wide spectrum of diseases. When any step of the clearance process fails - whether due to overwhelming apoptotic burden, molecular interference with recognition signals, or an inhospitable tissue environment - the consequences can be deleterious. Accumulating apoptotic cells induce secondary necrosis and the release of excessive DAMPs that perpetuate inflammation and tissue damage 30. Moreover, phagocytes with dysregulated efferocytosis can adopt aberrant phenotypes, such as overly immunosuppressive or foam-cell states, to promote disease development 5, 31. In this section, we introduce the pathological role of dysregulated efferocytosis in diverse diseases.

### 1.4. Autoimmune diseases

Autoimmune diseases are serious chronic disorders that arise from loss of self-tolerance to self-antigens, leading to persistent inflammation and multiorgan tissue damage 32. Defective efferocytosis has been associated with many autoimmune diseases, such as systemic lupus erythematosus (SLE). Patients with SLE exhibit large numbers of apoptotic bodies in tissues and circulation that are not properly cleared 33. These remnants release nuclear autoantigens (DNA, histones, etc.) that drive autoantibody production and inflammatory cascades 34. Multiple efferocytosis defects have been reported in lupus, such as macrophage-intrinsic dysfunction and the presence of autoantibodies against efferocytosis receptors 33. Lupus patients often have antibodies against C1q and other opsonins involved in AC clearance. Genetic deficiencies in complement components or bridging molecules (C1q, MFG-E8, MerTK/Axl/Tyro3) are risk factors for lupus, underscoring the key role of efferocytosis in preventing autoimmunity 35. Defects in efferocytosis during lupus chronically activate the immune system, breaking self-tolerance, inducing secondary necrosis and frustrating phagocytes in multiple tissues (e.g., kidneys).

In addition, a similar theme has been found in other autoimmune conditions, such as rheumatoid arthritis (RA). For instance, in the RA state, an imbalance of macrophage phenotypes in the joint leads to insufficient clearance of apoptotic neutrophils in the inflamed synovium, and the domination of pro-inflammatory macrophages and defective efferocytosis lead to the accumulation of cell debris and inflammatory cytokines in the local microenvironment, thereby perpetuating the degree of synovial inflammation and pannus formation 36. These reports suggest that defective efferocytosis can break immune tolerance and fuel chronic inflammation across diverse forms of autoimmune diseases.

### 1.5. Cardiovascular diseases

Atherosclerosis is a chronic inflammatory disease of medium- and large-sized arteries that affects millions of people worldwide 37. Although its mechanism is complicated, impaired clearance of ACs in atherosclerotic plaques has been proposed as a key driver of lesion progression 5. During early atheromas, macrophages efficiently phagocytose apoptotic lipid-laden foam cells. However, as plaques enlarge, macrophages become dysfunctional due to oxidative stress, cholesterol overload, and inflammatory cytokines. Multiple mechanisms underlie efferocytosis defects in advanced plaques, such as ADAM17/10-mediated shedding of MerTK, impaired cholesterol efflux because of reduced ABCA1, and the upregulation of the “don't-eat-me” signal CD47 on atheroma cells 7. The net result is defective efferocytosis: apoptotic foam cells accumulate in the plaque's core instead of being cleared. These uncleared corpses undergo secondary necrosis, forming a lipid-rich necrotic core that expands over time and destabilizes the plaque fibrous cap 5, potentially triggering acute thrombosis, heart attack or stroke 38, 39. Human studies of advanced plaques have found abundant apoptotic debris and markers of impaired efferocytosis (e.g., elevated lesional CD47 and soluble MerTK reflecting MerTK receptor cleavage) 7, 40. Thus, insufficient efferocytosis in plaques is considered a “linchpin” of atherosclerotic progression and instability. Conversely, enhancing efferocytosis reduced plaque size and promoted a more stable phenotype (with smaller necrotic cores and thicker fibrous caps) 41.

### 1.6. Neurodegenerative diseases

Neurodegenerative diseases, such as Alzheimer's disease (AD), are progressive, age-associated disorders characterized by neuronal loss and the accumulation of misfolded protein aggregates, leading to cognitive and functional decline 42. In the central nervous system, microglia serve as the major resident phagocytes responsible for clearing apoptotic neurons, synapses, and protein aggregates 43. However, microglial efferocytosis can become impaired or overwhelmed in neurodegenerative diseases. For instance, in AD brains, there is an accumulation of apoptotic or damaged neurons and excessive deposition of amyloid-β plaques, partly because microglia fail to effectively engulf and dispose of this debris 44. This may be due to chronic microglial activation (skewing them to a pro-inflammatory state with reduced phagocytic capacity), genetic factors (many AD risk genes, such as TREM2, are linked to microglial phagocytosis function), or competitive binding of soluble factors that block the eat-me signals 45. As a result, uncleared apoptotic cells and protein aggregates drive local inflammation and neurotoxicity in a vicious cycle to accelerate neurodegeneration. Moreover, in other neurodegenerative contexts (e.g., Parkinson's disease, ALS), evidence of defective clearance of dead neurons and myelin debris has been reported 46, 47. Interestingly, if microglia are overactivated in certain contexts, they might also phagocytose stressed but viable neurons (a phenomenon observed in some models), indicating that proper calibration of efferocytosis is vital for maintaining neural homeostasis.

### 1.7. Inflammatory disorders

In the conditions of acute inflammation or tissue injury, timely efferocytosis is also critical for resolution. For example, in lung inflammation, such as acute respiratory distress syndrome (ARDS), massive neutrophil influx into the lungs followed by delayed neutrophil apoptosis and heightened formation of neutrophil extracellular traps (NETs), whereas impaired efferocytosis and NET clearance by phagocytes can exacerbate lung injury 48, 49. Consistently, bronchoalveolar lavage fluid from ARDS patients *reduces* alveolar macrophage efferocytosis *ex vivo*, supporting a compartmentalized defect in corpse clearance within the lung 50. These abnormalities favor the persistence of neutrophils and the propagation of hyperinflammation (cytokine storm-like state) that enhances alveolar-capillary damage 51. Similar findings are also found in sepsis, where a dysregulated immune response can suppress macrophage function (sometimes called “immune paralysis”), leading to reduced clearance of microbes and apoptotic cells 52. In the severe trauma or burn state, the inflammatory response can likewise overshoot, and if massive apoptotic immune cells and apoptotic cells cannot be cleared, systemic inflammatory response syndrome (SIRS) can result 53, 54. Together, efficient tissue injury repair requires a switch from pro-inflammatory state to pro-resolving state, while defective efferocytosis tips the balance toward sustained inflammation and organ dysfunction.

### 1.8. Cancers

Disordered efferocytosis also plays a crucial role in the progression of multiple types of cancers because tumor cells often coopt efferocytic pathways as an immune evasion strategy 55. Many types of cancer cells (e.g., acute myeloid leukemia, breast cancer, and ovarian cancer) overexpress CD47 (an “don't eat me” signal) and other antiphagocytic ligands (e.g., CD24) to directly thwart macrophage-mediated cell clearance 21. Moreover, the tumor microenvironment (TME) is typically rich in apoptotic cells due to rapid cancer cell turnover or therapy-induced cell death, and tumor-associated macrophages (TAMs) avidly perform efferocytosis of these cells. This would seem beneficial, but in fact, TAM efferocytic activity drives them toward an M2-like, immunosuppressive phenotype 56. Upon engulfing apoptotic tumor cells, TAMs secrete elevated immunosuppressive cytokines (e.g., IL-10 and TGF-β) and express immune checkpoint ligands, thereby establishing an immunosuppressive and tolerogenic milieu 57. Indeed, elevated MerTK activity in TAMs has been associated with poor prognosis across multiple cancers, such as hepatocellular carcinoma and colorectal cancers 58, reflecting enhanced efferocytosis and subsequent suppression of cytotoxic T-cell responses. In essence, tumors exploit efferocytosis by evading phagocytosis when viable through “don't-eat-me” signals, yet leveraging TAM-mediated clearance of dead cells to dampen inflammation. Therefore, disordered efferocytosis in the TME has been considered an “immune checkpoint” that might be targeted to improve anti-tumor immunity 59.

In brief, dysregulated efferocytosis has been involved in diverse diseases. In some cases (atherosclerosis, autoimmune diseases, chronic inflammatory disorders), insufficient efferocytosis results in toxic or immunogenic material to accumulate that drives disease progression. In other cases (cancer), excess or contextually inappropriate efferocytosis by tumor-associated phagocytes contributes to an immunosuppressive microenvironment (**Table 1**). These insights set the stage for therapeutic interventions aiming to modulate efferocytosis - either boosting it to enhance clearance and resolution or tuning it down/changing its consequences to promote immunity.

## 2. Current strategies for modulating efferocytosis

Given the critical role of efferocytosis in diverse diseases, researchers have begun exploring potential therapies for modulating efferocytosis. To date, some pharmacological approaches that aim to enhance efferocytosis (in contexts such as cardiovascular, autoimmune, or resolution of inflammation) or to inhibit efferocytosis checkpoints (in cancer) have been reported and achieve therapeutic effects to some extent. In this section, we outline the main strategies under investigation as well as their limitations that motivate new solutions (**Table 2**).

### 2.1 Small molecule drugs

Small molecule modulators (mainly chemical compounds) provide a bidirectional lever to tune efferocytosis either enhancement in chronic inflammatory settings or inhibition in tumors to counter immunosuppression. Pharmacologically enhancing efferocytosis promotes apoptotic debris clearance and inflammation resolution in atherosclerosis. For example, activation of nuclear receptors, such as LXR via synthetic agonists (e.g., GW3965, T0901317), can transcriptionally upregulate efferocytic machinery MerTK in macrophages and augment efferocytosis to dampen inflammation 68, 87. A PPARγ agonist (pioglitazone) was found to restore macrophage efferocytosis of apoptotic neutrophils and normalize the sterile inflammatory response in chronic granulomatous disease models *in vivo* 70. In addition, the natural alkaloid columbamine was shown to promote LC3-associated phagocytosis (LAP), thereby enhancing macrophage efferocytosis and accelerating resolution in a murine colitis model 71.

In contrast, efferocytosis in the tumor microenvironment can reinforce immune suppression to promote cancer progression, and pharmacologic inhibitors of efferocytosis (e.g., MerTK) are being developed to boost an-titumor immunity 72. In pancreatic ductal adenocarcinoma (PDAC) liver-metastasis models, an oral MerTK inhibitor (UNC2250, 10 mg kg⁻¹) was found to improve CD8⁺ T-cell function and reduce metastatic burden 73.

However, systemic administration of an efferocytotic inhibitor (e.g., MerTK) may increase the risks of autoimmunity and tissue injury, as evidenced by lupus-like disease in Mertk-deficient mice and exacerbated autoimmunity in TAM receptor knockout settings 33. Moreover, the specificity of these small molecule inhibitors still has concerns, and some previously used MerTK inhibitors display polykinase activity, raising the risks of off-target effects and immunotoxicity. For example, BMS-794833 was originally developed against MET/VEGFR2 yet potently inhibits MerTK 74. Additionally, broad stimulation of macrophages may induce systemic side effects, and LXR agonists frequently induce hepatic steatosis and hypertriglyceridemia, which has hindered clinical translation 88. In addition, pharmacokinetics and delivery remain practical hurdles; for example, drug penetration into certain tissues (e.g., solid tumors) is often suboptimal, targeting lesion macrophages in atherosclerotic plaques is difficult 89, 90, and these limitations might be resolved using nanomedicine-based platforms.

### 2.2 Biologics and cytokines

Biologic agents, such as monoclonal antibodies and Fc-fusion proteins, may offer complementary ways to modulate efferocytosis by either removing antiphagocytic brakes or supplying pro-engulfment cues. One of the primary “don't-eat-me” pathways is CD47-SIRPα, which tumor cells exploit to evade clearance 21. Multiple biological agents, such as anti-CD47 antibodies (e.g., magrolimab) and engineered SIRPα-Fc decoy receptors (e.g., TTI-621, ALX148/evorpacept), have been tested in clinical trials, as they may enhance macrophage recognition and engulfment of tumor cells 76, 77. In addition, biologics can promote efferocytosis by providing opsonins or mimicking endogenous bridging molecules. Recombinant MFG-E8 protein binds phosphatidylserine on apoptotic cells and tethers them to phagocyte integrins (e.g., α_v_β_5_), which can improve efferocytosis and reduce inflammation in models of sepsis and hemorrhagic shock 44, 66, 78. Cytokine-based strategies may indirectly enhance efferocytosis by reprogramming macrophages. For example, IL-13 might drive a macrophage IL-10/Vav1/Rac1 circuit that increases phagosome formation and AC uptake during resolution 79.

However, biologics or cytokine-based treatments still face some challenges and biosafety concerns. For example, anti-CD47 antibodies can cause severe anemia because CD47 is highly expressed on red blood cells, SIRPα-directed decoys may have fewer anemia events, and infusion-type reactions predominate 91-93. In addition, systemically administered antibodies or recombinant proteins also have suboptimal tissue penetration and cell-specific effects 94, 95. Durable use of these drugs may elicit anti-drug antibodies (ADAs) that lower drug exposure and attenuate efficacy 96. Systemic administration of cytokines (e.g., IL-4) has produced multisystem adverse events in early trials, possibly due to its overall immunosuppression, making it difficult to titrate the “just-right” dose that augments efferocytosis without off-target effects 97. Together, while biologics offer receptor-targeting ability and have produced important clinical leads, their delivery issues and biosafety profiles also need to be improved.

### 2.3 Gene therapy

Gene therapy and gene-modulation strategies are emerging tools to tune efferocytosis by augmenting the phagocytic machinery in phagocytes or removing the inhibitory signals on target cells. Although most applications remain preclinical, proof-of-concept has been established that restoring or engineering efferocytic receptors can enhance AC clearance and dampen inflammation 82. As a receptor-augmentation paradigm, gene replacement of phagocytic retinal pigment epithelium (RPE) restores outer-segment phagocytosis and improves retinal structure and function in rats, targeting the MERTK gene, which progressed to a phase I trial in 2016, suggesting that supplementing a phagocytic receptor can rescue defective clearance *in vivo* 81. While the 2016 Phase I trial demonstrated acceptable ocular and systemic safety, with transient visual improvements in a subset of patients, subsequent-phase peer-reviewed clinical evidence and longer-term efficacy data remain limited, leaving the magnitude and durability of clinical benefit to be established 98.

Conversely, *ex vivo* CRISPR ablation of CD47 on tumor cells can increase macrophage phagocytosis and augment antitumor immunity in mice, and studies indicate that ~80% CD47 suppression might be required for phagocytosis to outpace tumor growth 83, 84. *In situ* dual gene editing via CD47 knockdown plus IL-12 overexpression can reprogram tumor cells into immune-activating hubs, which suppress tumor growth *in vivo* by driving TAM re-education and boosting efferocytosis and antigen-presentation crosstalk 99. Editing the phagocyte checkpoint SIRPα (e.g., CRISPR knockout or RNA silencing) renders macrophages “hyper-phagocytic,” improving engulfment of tumor targets and enhancing the performance of engineered macrophage therapies in preclinical studies 84, 85.

Although these results are promising, gene therapy to modulate efferocytosis remains early-stage and faces several challenges. For example, commonly used gene vectors, such as AAV, lack intrinsic tropism for macrophages in defined tissues, making off-target transduction likely unless one leverages cell-specific promoters, engineered capsids, or ligand-decorated carriers 100, 101. These limitations might be partially resolved by using targeted nanocarriers; for example, it has been reported that collagen IV-targeted NPs can deliver payloads into atherosclerotic plaques, and pH-gated nanoplatforms selectively engage TAMs in acidic compartments 102, 103. The possible biosafety and immune concerns of gene therapy also require caution. Viral vectors (e.g., AAV) can provoke innate and adaptive immunity, and durable transgene expression *in vivo* might induce unanticipated consequences. Furthermore, ethical and regulatory considerations for human gene editing, such as *ex vivo* CD47 editing or highly targeted *in vivo* editing, add complexity and cost. These hurdles may limit their clinical translation despite encouraging preclinical results. Thus, it is urgent to develop next-generation strategies to achieve more precise, safe, and efficacious modulation of efferocytosis.

## 3. Nanomedicine-based strategies for precision modulation of efferocytosis

**Figure 2:**
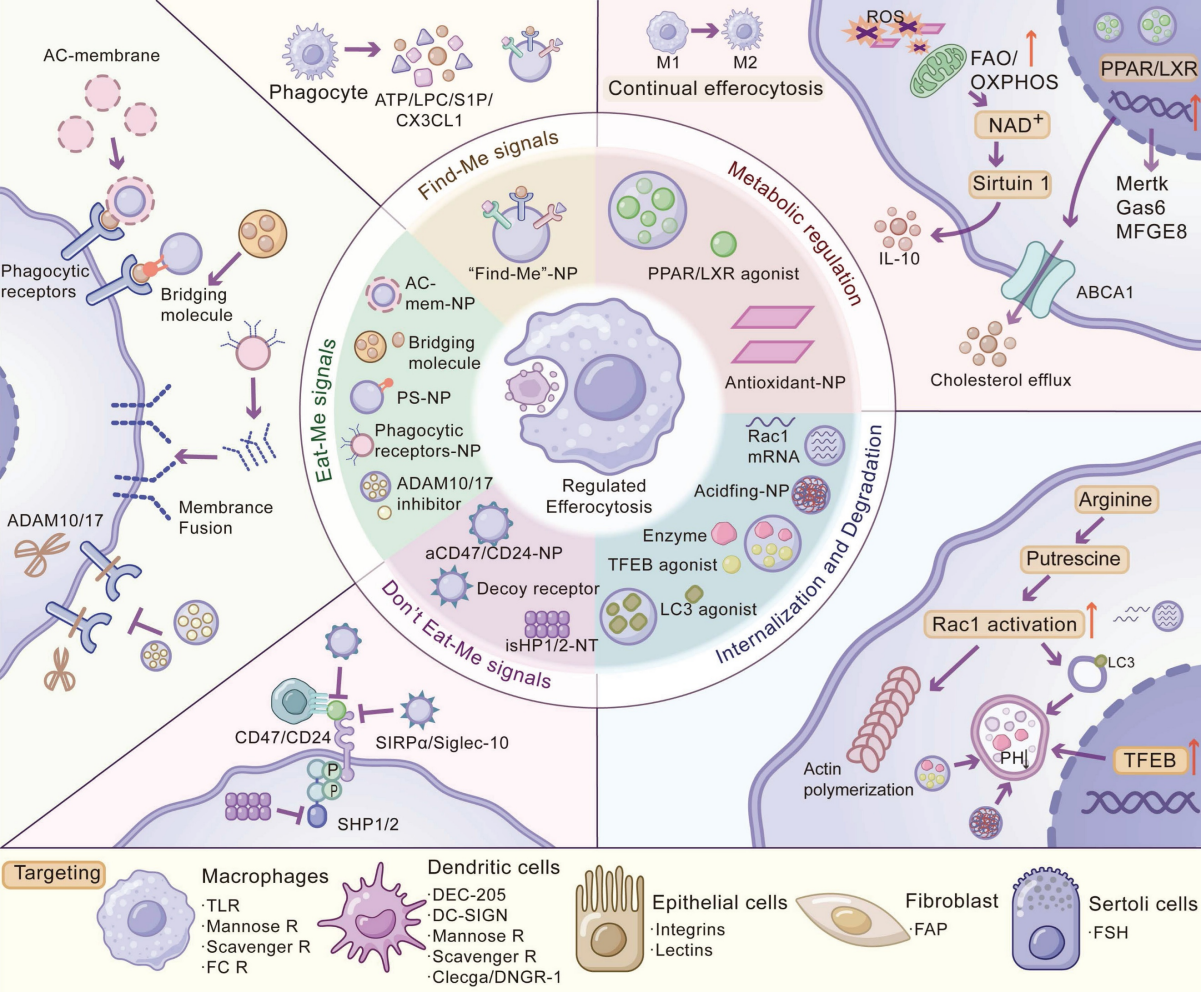
** Nanotherapeutic interventions across efferocytosis and cell-specific targeting.** The schematic centers on regulated efferocytosis and maps nanomedicine entry points across the process, with targeting cues summarized inline: nanoparticles that present or release ATP/UTP, LPC, S1P, or CX3CL1 (“find-me” NPs), recruit phagocytes to lesions; PS-decorated NPs and apoptotic-membrane-coated carriers mimic exposed PtdSer, while delivery of bridging molecules (Gas6, MFG-E8) or “receptor-NPs” upregulates/supplements MerTK and integrins, membrane fusion donates receptors, and ADAM10/17 inhibition prevents MerTK shedding to preserve uptake capacity; aCD47/aCD24-NPs multivalently block CD47-SIRPα or CD24-Siglec-10, decoy-receptor/peptide-modified NPs sequester inhibitory ligands, and macrophage-targeted SHP1/2 inhibition (iSHP1/2-NT) turns off intracellular brakes to restore phagocytosis; Rac1 mRNA or small-molecule boosters, LC3/LAP and TFEB agonists, acidifying materials, and lysosomal enzyme delivery enhance phagocytic-cup formation, efferosome maturation, lysosomal biogenesis, and efficient cargo digestion to prevent secondary necrosis; metabolic rewiring strategies—antioxidant nanozymes to temper excessive ROS, LXR/PPAR agonists to induce ABCA1-mediated cholesterol efflux and sustain MerTK, promotion of FAO/OXPHOS and the NAD⁺-sirtuin axis, and support of arginine→putrescine production to stabilize Rac1-actin dynamics—enable multi-round uptake and pro-resolving cytokines (e.g., IL-10); finally, receptor/ligand handles guide disease-site-specific delivery and combinations: macrophages (TLRs, mannose receptor, scavenger receptors, FcR), dendritic cells (DEC-205, DC-SIGN, mannose receptor, CLEC9A/DNGR-1), epithelial cells (integrins, lectins), fibroblasts (FAP), and Sertoli cells (FSH receptor). **Abbreviations:** PtdSer: phosphatidylserine; AC-mem: apoptotic cell membrane; LAP: LC3-associated phagocytosis; FAO: fatty acid β-oxidation; OXPHOS: oxidative phosphorylation.

Nanotherapeutics may offer a versatile platform to modulate efferocytosis with improved precision compared to conventional modalities. Nanocarriers can be functionalized to target phagocytic cells in specific tissues, maximizing the therapeutic index while minimizing systemic exposure 104. Such targeted delivery not only improves pharmacokinetics and lesion retention but also enables combinatorial therapy. For example, NPs can co-deliver a “don't-eat-me” signal inhibitor alongside a pro-engulfment agent, simultaneously blocking immune evasion and triggering efferocytosis in tumors. Moreover, nanocarriers can be engineered to mimic apoptotic cells by decorating their surface with phosphatidylserine or entire cell membranes, thereby enhancing their binding to macrophages and intrinsically activating anti-inflammatory, pro-resolving programs during uptake 105, 106. Stimuli-responsive nanoplatforms that selectively release drugs in specific pathological conditions (e.g., acidic pH, high ROS) can concentrate the therapeutic effect at disease sites while sparing healthy tissue 103. In this section, we discuss cutting-edge nanotherapeutic strategies, such as boosting “find-me” or “eat-me” signals, targeting phagocytic receptors and blocking “don't-eat-me” pathways, and tactics for delivering interventions to specific phagocyte subsets (**Figure 2, Table 3**).

### 3.1 Strategies for regulating efferocytotic signaling pathways

#### 3.1.1. Regulation of “find-me” signals

ACs actively release “find-me” cues, such as ATP/UTP, lysophosphatidylcholine (LPC), CX3CL1 (fractalkine), and sphingosine-1-phosphate (S1P), that establish chemotactic gradients to recruit phagocytes to sites of cell death. Based on this biology, nanoplatforms designed to augment or mimic find-me signaling and to enhance phagocyte recruitment have been reported. For example, NPs that present or release ATP in tumors increase intratumoral dendritic cell infiltration and potentiate antitumor immunity, which can be combined with checkpoint blockade therapy 107. In parallel, engineered efferocytosis-mimicking nanovesicles that co-display S1P (find-me) and phosphatidylserine (eat-me) can enhance macrophage recruitment and AC uptake, and S1P-enriched extracellular vesicles have also been shown to drive macrophage chemotaxis 108, 122. By strategies that aim to deliver or amplify “find-me” signals, nanoplatforms improve the homing ability of phagocytes to injured tissues. The chief advantage of this strategy is that it tackles the first limiting step in clearance: insufficient phagocyte presence.

However, limitations arise from the potential overstimulation leading to excessive inflammation if not precisely controlled. Another limitation is that simply recruiting phagocytes may not guarantee effective efferocytosis. For example, in atherosclerosis, simply recruiting more phagocytes to lesions might aid in clearing apoptotic cells, but it could also expand foam-cell burden and inflammation if not paired with restoration of lipid handling and efferocytic competence of those phagocytes 123. Thus, find-me enhancement is most useful when phagocyte scarcity is a major bottleneck (e.g., “cold” tumors). Overall, this strategy excels in enhancing phagocyte localization and can potently amplify immune responses, but it should be deployed with mechanisms to contain their effects spatially and paired with complementary strategies to ensure recruited phagocytes can perform their clearance function.

#### 3.1.2. Regulation of “eat-me” signals

The recognition (“eat-me”) phase of efferocytosis hinges on the exposure of phosphatidylserine (PS) on AC surfaces, which serves as a ubiquitous eat-me signal 124. Externalized PS binds to phagocyte receptors directly or via opsonins and bridging molecules (e.g., Gas6, Protein S, MFG-E8), triggering engulfment signaling 13. Nanotherapeutic strategies have increasingly focused on PS mimicry - decorating NP surfaces with PS or PS analogs to impersonate ACs and actively engage efferocytotic pathways. This approach exhibits two major advantages: (i) directing nanocarriers to phagocytes (macrophages, dendritic cells, etc.) and (ii) activating anti-inflammatory responses in those phagocytes akin to physiological efferocytosis 105, 106. For example, PS-decorated PLGA NPs showed increased accumulation in lung macrophages and reduced inflammatory readouts in LPS-induced acute lung injury 105. These findings align with earlier work showing that PS-presenting liposomes are preferentially taken up by macrophages and reprogram them toward a pro-resolving phenotype 65.

Besides PS coating, another approach is AC biomimetic NPs, including cell membrane-coated NPs and apoptotic body-like vesicles. By cloaking nanocarriers with the membrane of ACs, one can present a natural array of eat-me signals to phagocytes: engineered apoptotic vesicles derived from neutrophils (e.g., engineered neutrophil apoptotic bodies) can modulate skew macrophages toward a reparative, anti-inflammatory phenotype and reduce inflammatory cytokines in injury models, such as myocardial infarction, providing indirect evidence for improved efferocytosis 109. By impersonating the body's own apoptotic cells, such biomimetics ensure that they are rapidly bound and ingested by phagocytes.

To potentiate recognition, strategies involving bridging molecule enhancement have been reported. Mesenchymal stem cell-derived extracellular vesicles (MSC-EVs) enriched in GAS6 accumulated in the injured liver and activated MerTK signaling in hepatic macrophages, as evidenced by increased p-MerTK by Western blot, which in turn accelerated the clearance of apoptotic hepatocytes, quantified by fluorescence-based efferocytosis assays *in vitro*: pHrodo-labeled apoptotic cells were co-incubated with BMDMs and quantified by fluorescence microscopy and dampened inflammation 67; EVs with MFG-E8 expression also showed the ability to activate efferocytosis, with direct quantification of efferocytosis index via flow cytometry and confocal imaging 125. This illustrates how supplementing the local environment with bridging factors can kick-start latent efferocytosis pathways. Furthermore, an interesting design is to use bispecific constructs, named bispecific nanobioconjugates, that carry one moiety binding tumor cells and an “eat-me” ligand to promote macrophage phagocytosis 110. Such dual-targeted nanosystems can physically bring tumor cells and macrophages together to induce efferocytosis. This approach offers unique advantages in diseases characterized by defective recognition of apoptotic cells, such as atherosclerosis and autoimmune diseases. It should be noted that in neurodegenerative diseases, enhancing eat-me signals helps microglia recognize and clear dying neurons or protein aggregates. However, the overactivation of microglia could harm healthy neural connections; thus, deploying eat-me signal mimics must be done cautiously 126.

In addition, in the context of cancer, simply increasing efferocytosis via PS-coated nanoparticles could risk further polarizing TAMs toward an immunosuppressive state; encouragingly, innovative bispecific nanoconjugates offer a way to harness eat-me signals against live cancer cells. The challenges of this strategy include ensuring stable PtdSer exposure and potential saturation of phagocytic capacity, which could impair continual efferocytosis 27. In summary, nanotherapies that amplify eat-me signaling offer a highly biomimetic and modular route for phagocyte targeting and pro-resolution immunoregulation, but they require careful calibration of signal strength and companion immunostimulatory payloads in settings where immune stimulation is desired, particularly cancer.

#### 3.1.3. Regulation of phagocytic receptors

Regulation of (e.g., enhancing or inhibiting) phagocytic receptor activities has shown therapeutic promise. Phagocytic receptors such as MerTK are attractive checkpoints for therapeutic modulation. Nanotherapeutics provide multifaceted tools to enhance signaling through receptor delivery or upregulation 111 or inhibit or tune signaling at specific sites 127. By targeting these receptors, one can directly influence the cell-intrinsic “appetite” of phagocytes for apoptotic prey, either sharpening it to clear dangerous debris or damping it to allow controlled antigen release.

MerTK (Mer tyrosine kinase) has garnered particular interest as a nodal point in efferocytosis signaling and a drug target. In diseases such as atherosclerosis, enhancing MerTK-driven clearance can help resolve necrotic plaques, whereas in cancer, inhibiting MerTK on TAMs can prevent them from clearing tumor cell corpses and help restore antitumor immune responses 72, 111. Consequently, nanotherapeutics have been harnessed to modulate MerTK in both directions. A striking recent example is the targeted delivery of MerTK protein via a cell membrane-engineered NP. Hybrid nanovesicles coated with macrophage membranes overexpressing MerTK, these vesicles fused with lesional macrophages, effectively donating functional MerTK receptors to them, as confirmed by Western blot showing restored MerTK protein levels. Functionally, enhanced efferocytosis was directly quantified *in vitro* by flow cytometry (percentage of F4/80⁺ and CellTracker-labeled apoptotic-cell double-positive macrophages), complemented by confocal imaging-based assessment of macrophage uptake of fluorescently labeled apoptotic cells 111.

Also, the delivery of drugs that can enhance MerTK signals via nanocarriers is being explored. The nuclear receptor LXR drives MerTK expression and its ligands production 68, and delivery of an LXR agonist (T0901317) using PS-coated gold nanocarriers could increase macrophage MerTK expression and promote AC uptake 112. Another strategy is preventing the loss of MerTK activity. In the inflammatory state, enzymes (e.g., ADAM17) can cleave the ectodomain of MerTK off the cell surface and thus inactivate its efferocytic function 40, 64. To counteract this, neutrophil-mimicking nanoparticles (NPs) were constructed to deliver ADAM17 inhibitors for treating intracerebral hemorrhage, and such nanomedicine could inhibit ADAM17 and prevent local MerTK loss, as shown by Western blot (reduced ADAM17) and ELISA (decreased soluble MerTK), thereby boosting erythrophagocytosis (a form of efferocytosis) in the hemorrhagic brain with direct quantification *in vitro* by flow cytometry (fluorescent apoptotic RBCs co-incubated with BV2 cells) and visualization by confocal microscopy 113.

Conversely, strategies that aim to block phagocytic receptors can be beneficial in treating cancers. It has been found that TAMs suppress antitumor immunity through MerTK-dependent efferocytosis. Inhibiting MerTK signaling in the tumor microenvironment might convert immunosuppression into immunogenic responses 128. Nanoformulations of MerTK inhibitors (e.g., UNC2025, BMS-794833) allow them to be delivered specifically to TAM-rich regions, minimizing off-target effects 114, 129. Glycopolymeric NPs encapsulating a MerTK tyrosine kinase inhibitor (UNC2025) were shown to preferentially accumulate in TAMs and block efferocytosis to slow tumor growth 129. As a TAM family receptor, Axl (together with MerTK and TYRO3), has also been implicated in efferocytosis of cancer cells and can be targeted 69. TAM family receptors can be blocked using noncoding RNAs; for example, miR-34a suppresses AXL expression via targeting its 3′-UTR 130, and in a phase-1 trial, its liposomal mimic MRX34 achieved dose-dependent modulation of validated miR-34a targets (e.g., BCL2, CTNNB1, DNAJB1) with dexamethasone premedication, but it was halted for immune-mediated adverse events, underscoring the need for safer delivery 131. Likewise, delivery of AXL siRNA by melittin-derived peptide NPs reduces metastatic burden with limited toxicity in different cancer models, including ovarian and uterine models 132. Although these studies did not directly assess the impact of AXL blockade on efferocytosis, these promising findings may establish translatable precedents and motivate analogous designs that modulate AXL on tumor-associated phagocytes to influence efferocytosis in the tumor microenvironment. Besides, other phagocytic receptors, such as integrins, scavenger receptors, and immunoglobulin superfamily members like TIM-4, LILRB, and stabilin-2, are also vital for regulating efferocytosis, and whether they still have therapeutic potential is worthy of further investigation 24.

Rather than acting on signals emitted by apoptotic cells, this approach directly modulates receptors on phagocytes to reshape the execution of efferocytosis. Its comparative advantage is mechanistic precision: by upregulating or inhibiting a specific phagocytic receptor, one can augment or attenuate the phagocyte's intrinsic “appetite” for apoptotic targets in a controlled way. However, this strategy faces important limitations, including receptor-network complexity, potential compensatory pathways, and the challenges of targeting specific cell populations. First, the optimal receptor to target is disease dependent: in MASH mice, efferocytosis deficiency is mainly caused by TIM4 deficiency, while in atherosclerotic mice, efferocytosis deficiency in plaques is mediated by reduced MerTK 40, 133. Second, because phagocytes express numerous redundant receptors (MerTK, Axl, TIM-4, integrins, scavenger receptors, etc.) for apoptotic cells 24, focusing on a single receptor may be insufficient in some contexts or may require coordinated modulation of complementary steps. Safety also warrants careful consideration: systemic enhancement of receptor-mediated clearance could theoretically increase inappropriate or excessive removal 134. In this context, nanocarrier targeting offers a pragmatic mitigation strategy by confining receptor modulation to intended lesions and/or phagocyte subsets, thereby reducing systemic liabilities. Overall, nanocarrier-enabled phagocytic receptor modulation stands out for its directed mechanism and bidirectional tunability (up or down), but durable translational success will hinge on high-precision targeting and calibrated control of efferocytotic intensity to avoid unintended consequences at either extreme.

#### 3.1.4. Regulation of “don't eat me” signals

To avoid being mistakenly engulfed, healthy cells highly express surface proteins (“don't-eat-me” signals) that actively inhibit phagocytosis. For example, CD47 is ubiquitously expressed and engages SIRPα receptors on macrophages to deliver a potent anti-phagocytic signal. Many pathological cells, notably cancer cells, can exploit this pathway by overexpressing CD47 to cloake themselves from immune clearance 7, 135. Blocking CD47-SIRPα interactions by nanotherapies has emerged as a promising strategy to unleash phagocytes against targets, such as tumor cells or uncleared apoptotic debris. Anti-CD47-decorating NPs enable multivalent blockade of the CD47-SIRPα axis and co-delivery of chemotherapeutics (e.g., doxorubicin) to induce prophagocytic “eat-me” cues (e.g., calreticulin) and enhanced phagocytosis, as quantified *in vitro* by flow cytometry (percentage of CFSE⁺eFluor670⁺ double-positive BMDMs among total eFluor670⁺ BMDMs) 115. Similarly, anti-CD47-loaded calcium-carbonate NPs (aCD47@CaCO_3_) were incorporated into a sprayable fibrin gel, and the degradation of CaCO_3_ NPs in the acidic tumor microenvironment led to the release of anti-CD47 and reduced tumor recurrence in melanoma post-surgical resection models 136. Another interesting strategy is the “Trojan horse” NP. SHP1, a downstream phosphatase of the SIRPα signaling cascade, can transmit the “don't eat me” signal 137. Macrophage-targeting single-walled carbon nanotubes (SWNTs) were used to deliver SHP1 inhibitors 41, which reactivated macrophage efferocytosis in plaques, as demonstrated by direct quantification *in vitro* (flow cytometry-based efferocytosis assay) and *in vivo* (reduced apoptotic cell accumulation in plaques via histological analysis) and without causing anemia in atherosclerotic mice and large animal (porcine) models 9, 41. This exemplifies how nanotherapeutics can add a layer of specificity; unlike free anti-CD47 antibodies, nanotherapeutics can concentrate the anti-“don't-eat-me” effect only where it is needed (e.g., within TAMs or plaque macrophages).

Another approach is to use decoy receptors or peptides presented by NPs. A peptide derived from SIRPα's binding domain (Pep20) has been developed that binds CD47 without activating the inhibitory signal 138. Surface modification of Pep20 on metal-organic framework NPs loaded with STING agonists has been shown to have antitumor potential by enhancing macrophage phagocytosis without causing anemia 116. Functionalized NPs with fragments or membrane-presented forms of SIRPα may act as sponges, binding to CD47 on target cells and masking it. Representative samples include liposomes displaying the Fc-fused high-affinity SIRPα variant CV1 (Fc-CV1) on their surfaces 139; microwave-responsive Prussian blue NPs cloaked with macrophage membranes overexpressing SIRPα (SIRPα-M@nanoPB) 140; and EVs engineered to display SIRPα (EV-SIRPα) as nanocarriers 141.

Turning off inhibitory signals on phagocytes can convert them into more aggressive “hunters” of diseased cells. In addition to CD47, some other “don't eat me” signals are being targeted as well. CD24, which can bind Siglec-10 on TAMs, was recently identified as an antiphagocytic signal in multiple cancers, such as ovarian cancer 142 and hematological malignancies 143. Recently, CD24-targeting therapies have gained traction, and multiple anti-CD24 antibodies have displayed a role in enhancing macrophage phagocytosis in preclinical reports and are going into early clinical trials 144, 145. NP-based strategies can improve efficacy while reducing the side effects of antibody therapy and offer the potential for straightforward combinatorial treatment regimens through simple formulation adjustments. For instance, anti-CD24 antibodies conjugated to endocytic nanospheres have been developed to disrupt the CD24-Siglec10 axis, enhance macrophage phagocytosis and suppress tumor growth 117. Ongoing research is extending this paradigm to solid tumors, often combining CD47/CD24 blockade with other therapies (e.g., cytotoxic drugs or immune checkpoint inhibitors) for synergistic effects 146.

Blocking inhibitory phagocytic axes such as the CD47-SIRPα pathway has become a prominent therapeutic strategy; antibodies and SIRPα-based decoy proteins targeting this axis have entered clinical trials, supporting its translational relevance as a druggable target 147. The major limitation of this strategy is the potential for collateral damage to healthy cells that also express CD47 (notably red blood cells). Nanoparticle targeting offers a potential mitigation route by increasing local exposure in tumors or disease-relevant myeloid niches, thereby widening the therapeutic window and potentially reducing anemia risk. However, evidence for substantial mitigation of systemic toxicities remains largely preclinical and warrants rigorous clinical validation. Accordingly, in diseases requiring systemic therapy (e.g., hematologic malignancies), the application of this strategy demands careful optimization of its risk-benefit profile through dosing schemes, rational combinations, and proactive toxicity management 147. Overall, nanotherapeutic blockade of anti-phagocytic signals is most compelling in cancer and selected localized chronic diseases where local delivery or cell-directed targeting can enhance specificity. Furthermore, patient stratification (e.g., selecting tumors with high CD47 expression) may be critical to maximize benefit-risk. In disease without clear “don't-eat-me” signal dependence, the anticipated therapeutic benefit is likely limited, while risks such as anemia or other hematologic toxicities remain substantial.

#### 3.1.5. Regulation of internalization and degradation

After the recognition and binding stages, phagocytes can internalize the target cells and degrade them via phagolysosomes and lysosome routes 13. This stage can also be inefficient when phagocytes are “overloaded” and/or their lysosomal function is impaired, resulting in abortive efferocytosis, such as incomplete digestion and secondary necrosis 148. The small GTPase Rac1 governs actin polymerization during phagocytosis, and activation of Rac1 drives pseudopod extension and AC engulfment. Currently, nanotherapeutics are being developed to ensure that engulfment and processing proceed optimally. One emerging approach is to use macrophage-targeted NPs to modulate the RAC1-actin module that powers efferocytosis. For example, lipid nanoparticle (LNP)-mediated delivery of Rac1 mRNA to monocytes/macrophages could improve antifungal pathogen clearance *in vivo*, supporting the idea that boosting Rac1 can augment phagocyte internalization 118. Interestingly, NP itself might affect the Rac1 pathway due to its intrinsic properties, and synthetic silica NPs were shown to induce strong Rac1 activation and actin polymerization in macrophages 149. Efferocytotic macrophages can convert AC-derived arginine/ornithine to putrescine, which stabilizes Mcf2 mRNA and increases Rac1 activity to enable continual engulfment 29. In this aspect, macrophage-targeted nanotherapy may in turn activate the Rac1 signaling pathway to restore efficient internalization during efferocytosis.

Nanomedicine-based approaches for enhancing lysosomal degradation capacity have also been reported. In advanced atherosclerotic lesions, repeated uptake of ACs and lipids can induce macrophage lysosomal stress, which impairs lysosomal acidity and degradative capacity 150. Acidifying NPs, typically made of acidic or carboxyl-terminated polymers, can accumulate in endolysosomes and donate protons to re-acidify lysosomes and recover hydrolase activity 119. A complementary strategy is to drive lysosomal biogenesis. TFEB is the principal regulator of lysosomal biogenesis, and it activates the coordinated lysosomal expression and regulation (CLEAR) network to increase lysosomal abundance and acidification 151. TFEB agonists, such as trehalose, have been shown to induce lysosomal biogenesis and confer protection in disease models (e.g., atherosclerosis) 152. Nanocarrier-based approaches can further enhance efficacy via targeted delivery of TFEB inducers that promote TFEB nuclear translocation, increasing their intralysosomal effective concentration and amplifying CLEAR-mediated degradation. Beyond small molecules, targeted lysosome enzyme supplementation might be feasible via delivery routes based on mannose- or mannose-6-phosphate, suggesting a potent path for macrophage-directed hydrolase augmentation 153.

The process of AC process and disposal is critical for sustaining efferocytosis. LC3-associated phagocytosis (LAP) is a non-canonical pathway in which LC3 is conjugated to single-membrane phagosomes, thereby accelerating fusion with lysosomes, and boosting LAP might couple phagosome maturation with efficient lysosomal clearance. Small molecule compounds (e.g., columbamine) can enhance LAP, and macrophage efferocytosis has been reported: researchers have performed direct *in vivo* quantification of systemic efferocytosis by measuring the CMFDA-positive apoptotic-cell-derived signal in the liver and spleen by flow cytometry and in tissues using TUNEL labeling of ACs together with macrophage staining to assess *in situ* efferocytosis 71; nanoformulation of such agonists is a plausible strategy to enhance LAP signaling in phagocytes of lesion sites. Unlike canonical autophagy, LAP is mainly independent of the AMPK-mTORC1-ULK1 nutrient-sensing axis 25. Although mTOR inhibitors or general autophagy inducers may enhance the overall lysosomal capacity 154, they might not be equated with LAP activation for efferocytosis intervention, given LAP's mTOR-independent control logic. Together, by fine-tuning the intracellular phase of efferocytosis, these nanomedicines may prevent secondary necrosis and pro-inflammatory leakage.

Augmenting the internalization and degradation steps of efferocytosis may be particularly beneficial in diseases where phagocytes are overwhelmed by apoptotic or necrotic debris and post-engulfment processing becomes rate-limiting, such as advanced atherosclerosis and neurodegenerative disorders 5, 155. In cancer, however, the same strategy is highly context-dependent because efficient efferocytosis by tumor-associated macrophages often induces IL-10/TGF-β-linked immunosuppressive programs that can dampen antitumor immunity. Accordingly, for therapies aiming to enhance antitumor immunity, accelerating macrophage internalization/degradation is usually not favored in cancer. A key limitation of this strategy is sustainability and cellular capacity: accelerating internalization/degradation without concomitant support for metabolic and recycling programs may impose lipid, lysosomal and energetic burdens, leading to a transient increase in clearance followed by a functional decline in continual efferocytosis 156. Thus, interventions that boost this phase may be more durable when paired with support for cholesterol homeostasis and mitochondrial adaptation.

#### 3.1.6. Metabolic regulation for continual efferocytosis

Phagocytosis of ACs imposes significant metabolic demands on phagocytes, as a bolus of AC-derived materials, such as proteins, lipids, cholesterol, and nucleic acids, must be efficiently processed 27, 28. When this metabolic adaptation is perturbed, macrophages lose capacity for subsequent rounds—internalizing one apoptotic cell but failing on the next—resulting in uncleared corpses and impaired resolution 29. Strategies of metabolic regulation can sustain the ability of phagocytes to perform continual efferocytosis. One of the prominent targets is the LXR-PPAR pathway, since LXR upregulates ABCA1/G1 transporters for cholesterol efflux and preventing lipid overload 157. Nanotherapeutics delivering cholesterol exporters or agonists of nuclear receptors (LXRα/β or PPARγ) have been utilized. By activating LXR/PPAR pathways, these treatments upregulate ApoE and ABCA1/G1 transporters to efflux excess cholesterol and induce an anti-inflammatory gene profile, effectively rejuvenating macrophages' ability to continue efferocytosis 120, 158, 159. Designing LXR-agonist nanocarriers for macrophage receptor uptake yields selective macrophage delivery and circumvents hepatocyte uptake, thereby effectively blocking the effect of LXR agonists on hepatic adipogenesis 160, suggesting the superiority of cell-specific metabolic therapy.

Macrophages metabolize AC corpse-derived arginine via arginase-1 (Arg1) and ornithine decarboxylase (ODC) into polyamines (e.g., putrescine) 29, which can sustain the Rac1 signaling required for actin cytoskeletal rearrangements in continual efferocytosis 29. Therapeutic enhancement of polyamine production or supplementation with exogenous polyamines has been shown to restore efferocytic capacity in macrophages facing heavy apoptotic loads 29. To overcome the rapid turnover and short half-life of polyamines *in vivo*, NP-based formulations of polyamines or their precursors are being explored. Polymeric microparticles co-delivering the polyamine spermine and α-ketoglutarate, an intermediate of the tricarboxylic acid (TCA) cycle, have been found to reprogram macrophages toward a pro-resolving phenotype that is conducive to efferocytosis 156, 161. By reprogramming cellular metabolism, such nanomedicines ensure that phagocytes have sufficient energy and biosynthetic capacity to perform continual efferocytosis.

Macrophages undergoing efferocytosis shift toward enhanced oxidative metabolism (increased fatty acid β-oxidation and OXPHOS) to supply ATP and pro-resolving molecules (e.g., IL-10) production 156, 162. However, continual efferocytosis requires tight control of mitochondrial membrane potential and reactive oxygen species (ROS) 163. Antioxidant nanozymes that scavenge excess ROS preserve mitochondrial integrity and sustain or reactivate efferocytosis *in vivo* 121. For example, cerium oxide nanozymes cloaked with apoptotic neutrophil membranes reduced macrophage oxidative stress and reactivated continual efferocytosis, thereby accelerating inflammatory resolution and bone repair 164. During efferocytosis, the NAD⁺-SIRT1 axis is required for robust IL-10 production 162, 165, and strategies that aim to bias macrophages toward an enhanced oxidative phenotype by activating PPAR or AMPK pathways may boost continual efferocytosis and inflammation resolution. Nanotherapeutics can be designed for targeted delivery of metabolic drugs, nutrient precursors, or instructive signaling molecules to boost mitochondrial fatty acid β-oxidation, polyamine biosynthesis, or cholesterol efflux.

The chief advantage of metabolic interventions is their potential to restore and sustain phagocyte “endurance”, thereby supporting long-term clearance of apoptotic cells and inflammatory resolution beyond a single engulfment event. Because these approaches frequently rely on transcriptional programs and organelle homeostasis (e.g., nuclear receptor-driven cholesterol efflux pathways), metabolic rewiring may take time and is therefore most pertinent to chronic settings featuring phagocyte overload or fatigue, such as atherosclerosis and chronic inflammatory/degenerative diseases. However, the ubiquity of metabolic pathways mandates tight specificity: LXR/PPAR signaling and redox balance operate across many cell types, so nontargeted modulation can dilute on-site efficacy and introduce systemic liabilities (for example, LXR agonist-linked hepatic lipogenesis and hypertriglyceridemia) 88. Targeted delivery has therefore been proposed to avoid systemic metabolic side effects and expand the therapeutic window. In cancer, whether to promote macrophage cholesterol efflux is context dependent: in a metastatic ovarian cancer model, cholesterol efflux has been reported to drive tumor-promoting TAM programs 166. Consistently, efferocytosis itself can reprogram macrophages toward immunosuppressive states.

Conversely, in other tumor settings, increased macrophage ABCA1 activity has been reported to reduce efferocytosis without influencing phagocytosis, illustrating that “ABCA1/LXR-linked” programs can also align with antitumor outcomes depending on the mechanism and cellular state 167. Importantly, ABCA1 has been summarized as a facilitator of efferocytosis-related signaling in nontumor contexts, underscoring the need to choose directionality (promote versus restrain efflux programs) based on mechanism-of-action goals, tumor context, and TAM lineage/state. In summary, metabolic reprogramming strategies can prolong and potentiate the efferocytosis process by addressing the less visible but crucial constraint of phagocyte endurance; they may form a backbone for chronic inflammatory disease therapy and synergize with direct efferocytosis enhancers (find/eat-me cue modulation or receptor/engulfment agonism) to improve both uptake and processing.

### 3.2 Strategies for targeting diverse phagocyte types

In addition to the strategies that focus on which signals or pathways to modulate, an equally important consideration is which phagocytic cells are being targeted. Different phagocyte types and/or their tissue locations require different delivery strategies to achieve specific and efficient modulation. Nanotherapeutics can be engineered for targeting particular cell types by leveraging unique surface markers, tropism, or anatomical access routes 168-170. In this section, we briefly discuss the strategies tailored to professional phagocytes (e.g., macrophages, DCs) and nonprofessional phagocytic cells (e.g., epithelial cells, fibroblasts, Sertoli cells).

#### 3.2.1. Professional phagocytes

##### Macrophages

Macrophages are primary effectors of efferocytosis in most tissue types 171. It is well documented that systemically administered NPs are mainly sequestered by the mononuclear phagocyte system, particularly Kupffer cells in the liver and red pulp macrophages in the spleen 172. Instead, NPs with ligand decoration can actively target different macrophage subsets. M2-like macrophage subsets, such as TAMs, highly express the mannose receptor (CD206), and mannose-decorated NPs showed increased uptake in these macrophages 173. Folate receptor-β (FRβ) is often upregulated on activated macrophages in inflamed tissues and can be targeted via folic acid modification. For example, [^18^F]fluoro-PEG-folate selectively images FRβ^+^ synovial macrophages in rheumatoid arthritis 174. Antibodies or antibody fragments to macrophage scavenger receptors (MARCO, SR-A) or integrins (such as CD11b) have been attached to NP surfaces to preferentially target macrophages in plaques or sites of infection 175, 176.

The biomimetic/signal mimicry strategy involves wrapping NPs with signal molecules or membranes from cells that macrophages routinely interact with. For instance, PS-mimicking nanotherapeutics and apoptotic body biomimetics have enhanced uptake by macrophages 105, 177. Coating PLGA NPs with bacterial outer membrane vesicles (OMVs) enables prolonged retention in the peritoneal cavity and efficient macrophage uptake after ip administration 178. Additional strategies employ the concept of “hitchhiking” on circulating monocytes or macrophages. For example, loading of NPs into autologous monocytes *ex vivo* followed by reinfusion or injecting NPs endowed with cell-targeting specificity into the body, enabling them to attach to or be internalized by target cells, relies on these cells' natural homing to inflammation sites to ferry the therapy directly for targeting macrophages in injured tissues 179. The route of administration may also affect the macrophage-targeting effect. Intravenous injection tends to favor uptake by liver and spleen macrophages 180; inhalation effectively delivers NPs to alveolar macrophages in the lung 181.

##### Dendritic cells

Dendritic cells (DCs) are also important professional phagocytes that specialize in antigen processing and presentation 171. Nanotherapeutics targeting DCs are especially useful for immunotherapies (vaccines, cancer immunotherapy) and for inducing tolerance in autoimmune disorders 182, 183. Unlike macrophages, many DCs reside in or traffic through lymphoid organs and thus require delivery to lymph nodes or skin/mucosal surfaces. NP size influences the efficiency of DC targeting; after subcutaneous administration, small NPs (~20-200 nm) can drain via lymphatics to reach lymph node-resident DCs, while larger particles (~500-2000 nm) tend to remain at the injection site and be taken up by skin DCs that subsequently migrate to the node 184. A wide array of DC surface receptors, such as C-type lectins (e.g., DEC-205, DC-SIGN, mannose receptor), Fc receptors, and scavenger receptors, have been exploited for broad DC-targeting delivery 185. For example, PLGA NPs functionalized with an anti-DEC-205 antibody delivered melanoma antigens directly to DEC-205⁺ DCs, improving cross-presentation and boosting CD8⁺ T-cell responses compared with nontargeted formulations in preclinical models 186. Mannan-coated antigen-loaded NPs have also been used for targeting DCs and have induced tolerogenic programs in autoimmune settings 187. DC subsets might be targeted via specific surface receptors; for example, conventional dendritic cell 1 (cDC1) can be targeted via an XCL1-XCR1 axis 188. CLEC9A-targeted nanovaccines exploit DNGR-1-mediated endocytosis in cDC1s to boost cross-presentation and antitumor efficacy 189.

In brief, DC-targeting nanotherapeutics require the comprehensive application of strategies, such as NP size and specific surface labeling (CLEC9A, DEC205, etc.). These interventions can boost the role of DCs in efferocytosis - whether to maintain self-tolerance (clearing apoptotic self-cells without inflammation) or to enhance immunity (efficiently processing tumor cell corpses into antigens for T cells). The result is a more controlled and effective linkage between innate clearance and adaptive immune activation.

#### 3.2.2. Non-professional phagocytes

##### Epithelial cells

Epithelial cells in diverse organs can function as non-professional phagocytes, engulfing ACs to maintain local tissue homeostasis 1. For instance, retinal pigment epithelial (RPE) cells in the eye can phagocytose shed photoreceptor outer segments, mammary epithelial cells (MECs) in the postpartum gland can phagocytose apoptotic cells during involution 190, 191, and lung airway epithelial cells engulf ACs via Rac1-dependent signaling 192. Despite being less efficient than macrophages, these epithelial efferocytes also produce anti-inflammatory mediators that restrain inflammation 1. Nanotherapeutic strategies are being designed to deliver payloads specifically to epithelial surfaces in the eye, mammary gland, respiratory tract, and intestine. One approach is ligand-directed targeting of epithelial receptors. Integrin-binding peptide (e.g., RGD)-decorated NPs showed enhanced binding and uptake by epithelial cells in target tissues 193. In the intestine, NPs functionalized with lectins (such as Aleuria aurantia or tomato lectin) can bind to glycosylated apical proteins on M cells and enterocytes, boosting transcytosis across the epithelium 194. Mucosal surfaces in the respiratory and ocular tract can be targeted via mucoadhesive lectins or antibodies to cell-specific surface proteins, which ensure that NPs can arrive at epithelial cells rather than being cleared 195. Another strategy is topical or local delivery of complement receptor targeting. For example, aerosolized lipid-NPs can accumulate on airway epithelium, intravesical NPs have increased bladder epithelial retention, and oral/rectal nanoformulations enhance gastrointestinal epithelial access 196-198.

##### Fibroblasts

Fibroblasts, classically viewed as connective-tissue builders, nonetheless display certain non-professional phagocytic activity, including engulfment of ACs and matrix debris during tissue remodeling and repair 199. Fibroblasts can internalize extracellular collagen via endocytic routes (e.g., uPARAP/Endo180) for lysosomal degradation, underscoring a capacity for matrix “phagocytosis-like” clearance 200. In chronic fibrosis and the tumor stroma, activated fibroblasts (myofibroblasts or CAFs) are abundant and can profoundly shape immune infiltration and the local resolution milieu, indirectly influencing efferocytosis 201. Fibroblast activation protein (FAP), which is highly expressed on activated fibroblasts in fibrotic and neoplastic lesions, enables selective delivery. FAP-targeted NPs have been shown to bind FAP^+^ fibroblasts in imaging and therapeutic contexts 202, 203. Given the documented fibroblast uptake of apoptotic bodies and ECM, augmenting these clearance pathways with fibroblast-targeted NPs represents a forward-looking avenue to aid tissue cleanup when professional phagocytes are overwhelmed.

##### Sertoli cells

Sertoli cells are specialized non-professional phagocytes that continuously engulf apoptotic germ cells and residual bodies during spermatogenesis; this efferocytosis is indispensable for testicular homeostasis and fertility, in which TAM receptors (e.g., MerTK/AXL) play a central role 204. However, sertoli cells are located behind the blood‒testis barrier (BTB), which limits systemic NP access and poses a major delivery challenge 205. Nanomedicine may offer potent strategies to surmount the BTB for testicular drug delivery, and certain nanosystems (e.g., branched gold-copper nanocrystals) have been shown to penetrate into seminiferous tubules 206. Nanomaterial design also influences BTB traversal, and optimizing size, shape and surface functionalization (e.g., FSH-peptide targeting of Sertoli cells) might enhance testis-specific delivery 207, 208. Moreover, local intratesticular (intratubular) injection bypasses the BTB entirely, depositing nanocarriers directly into seminiferous tubules and leveraging Sertoli cell transcytosis to shuttle them across compartments 209. By coupling ligand modification with local delivery, nanocarriers may deliver payloads that augment TAM signaling (e.g., MerTK ligands) and potentiate Sertoli efferocytosis, offering a rational strategy for male infertility or orchitis.

## 4. Therapeutic effects of efferocytosis-modulating nanomedicines

**Figure 3:**
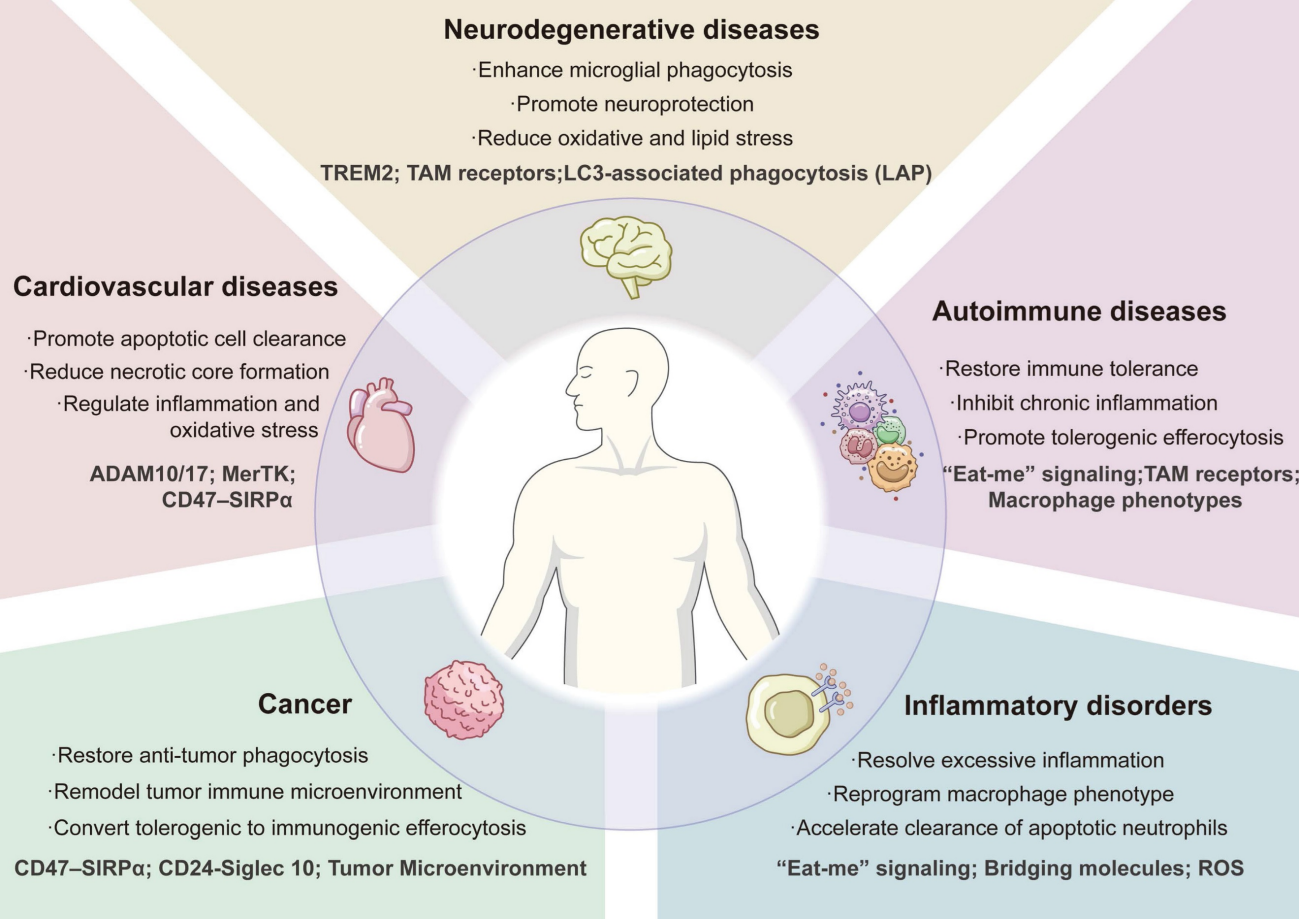
** Efferocytosis-modulating nanotherapeutics across disease settings: goals and actionable pathways.** The radial layout summarizes therapeutic objectives and tractable pathways for efferocytosis-centered nanomedicine across five disease classes. **Abbreviations:** TAM: Tyro3/Axl/MerTK receptor family; LAP: LC3-associated phagocytosis.

Inefficient clearance of apoptotic cells is a pathogenic feature in many conditions, from chronic inflammatory disorders where uncleared debris drives inflammation to tumors where phagocytic clearance paradoxically supports immune evasion. Nanotherapeutics designed to enhance or recalibrate efferocytosis are emerging as innovative interventions across these diverse pathologies. In this section, we discuss the therapeutic role of efferocytosis-modulating nanomedicines in diverse diseases, such as cardiovascular disease, autoimmune disease, neurodegenerative disease, inflammatory disorders, and cancers (**Figure 3, Table S1**).

### 4.1 Cardiovascular diseases

Atherosclerotic plaques are characterized by accumulated apoptotic foam cells and a large necrotic core. In advanced lesions, macrophage phagocytic receptors such as MerTK are often cleaved or downregulated, whereas “don't-eat-me” signals (e.g., CD47) on dying foam cells are upregulated, collectively impairing AC clearance. Thus, efferocytosis-modulating nanomedicines for atherosclerosis focus on restoring phagocyte recognition and engulfment capacity, blocking inhibitory signals in plaques, and delivering pro-resolving cues to the inflammatory milieu. One strategy is to upregulate phagocytic receptors on lesional macrophages. For example, engineered hybrid-membrane nanovesicles (HMNVs) were constructed by fusing membranes from MerTK-overexpressing macrophages and transferrin receptor-overexpressing HEK293T cells, incorporating DOPE polymers and superparamagnetic iron oxide for magnetic navigation. In diabetic ApoE⁻/⁻ mice, HMNVs preferentially accumulate in the aorta to quell plaque inflammation under an external magnetic field and then fuse with recipient macrophage membranes and donate functional MerTK to restore efferocytosis 111. This “receptor delivery” approach directly tackles the MerTK insufficiency known to exacerbate plaque necrosis. Macrophage-targeted delivery of liver X receptor (LXR) agonists into atherosclerotic plaque macrophages via polymeric PLGA-PEG nanoparticles (NP-LXR) increased cholesterol-efflux machinery (ABCA1/G1) and MerTK expression to damp inflammatory signaling 156; sHDL NPs carrying LXR agonists (T0901317) enhanced atheroma targeting, boosted macrophage cholesterol efflux, and inhibited lesion progression in ApoE⁻/⁻ mice 210.

A complementary tactic addresses the prevalent “don't-eat-me” signal in plaques. Blockade of CD47 via antibodies can restore macrophage efferocytosis 7, but systemic anti-CD47 administration causes severe hematologic toxicity because of the high expression of CD47 on RBCs. To overcome this issue, macrophage-specific nanocarriers have been pursued; macrophage-specific single-walled carbon nanotubes (SWNTs) loaded with an SHP-1 inhibitor (downstream of CD47/SIRPα signaling) selectively target lesional phagocytes, reactivate efferocytosis, and reduce the necrotic core in ApoE^⁻/⁻^ mice without hematologic toxicity 41. Moreover, this SHP-1-inhibitor-based nanomedicine can reduce AC accumulation and intraplaque inflammation without anemia in a porcine model of atherosclerosis, highlighting its translational potential 9. Therefore, cell-specific checkpoint blockade via a nanoplatform can capture the pro-efferocytic benefit of CD47 inhibition while avoiding anemia and systemic immunosuppression.

In addition to the efferocytotic receptor and checkpoint, pro-resolving mediators or reprogramming cues can also be delivered by nanoplatforms to correct the inflammatory milieu that impairs macrophage efferocytosis. For example, ROS-responsive PEGylated black phosphorus nanosheets (BPNSs) decorated with the macrophage stabilin-2-targeting S2P peptide were used to deliver resolvin D1 (BPNSs@PEG-S2P/R), which can accumulate in lesional macrophages, release RvD1, and scavenge ROS, thereby reducing plaque burden in ApoE⁻/⁻ mice 211. Nanotherapies that can modulate macrophage phenotype switching to enhance efferocytosis have also been reported. CpG-conjugated silver NPs (CpG-AgNPs) enhanced macrophage efferocytosis of CD47-positive apoptotic and foam cells via TLR9-mediated metabolic reprogramming (boosting fatty acid oxidation); the AgNP core also contributed to lesion control due to its intrinsic anti-inflammatory effect in ApoE⁻/⁻ mice 212. Another biomimic nanoformulation composed of atorvastatin (AT) and metformin (Met)-loaded hyaluronic acid-coated and macrophage membrane-camouflaged NPs (HA-M@P@(AT+Met)) could target lesional macrophages and shift them toward M2-like phenotypes with enhanced efferocytosis via the ERK5-MerTK pathway, thereby reducing necrotic cores and stabilizing plaques *in vivo* 213. This strategy that aims to deliver combination therapies in a targeted fashion underscores the combinatorial power of nanotherapeutics in tackling multiple facets of efferocytosis dysfunction simultaneously.

### 4.2. Autoimmune Disease

Autoimmune diseases are commonly characterized by a breakdown in self-tolerance and chronic inflammation, and defective efferocytosis has been recognized as a critical pathological factor. Nanomedicines that aim to enhance the clearance of apoptotic debris or modulate phagocytes toward tolerance are therefore of great interest in such diseases. Leveraging the natural “eat me” signal PS has emerged as an attractive strategy. By presenting PS on the nanocarrier surface, it engages phagocytes and induces anti-inflammatory, tolerogenic signaling during AC uptake 214. An efferocytosis-informed nanoimitator (EINI) whose core complexes siIRF5 with a metformin-Zn²⁺ agent to modulate macrophage metabolism is cloaked by a ROS-responsive PS corona and is decorated with low-molecular-weight heparin (LMWH, antagonize P-selectin) for lesional targeting. In rheumatoid arthritis (RA) mice, EINI treatments could reprogram synovial inflammatory macrophages and mitigate autoimmune joint pathology 62. Likewise, PS-liposome-coated gold nanocages carrying LXR agonists (T0901317) upregulated MerTK on macrophages and enhanced AC clearance while alleviating kidney damage in systemic lupus erythematosus (SLE) models 112. Apoptotic-mimicking NPs have also been utilized for antigen-specific tolerization. PS-coated liposomes encapsulating insulin peptide were engulfed by macrophages, which then presented the antigens to lymphocytes in a tolerogenic context. This nanotherapy shifted macrophages toward an anti-inflammatory phenotype and increased Treg responses in non-obese diabetic mice 215. Biomimetic NPs combining PS-mediated tolerogenic signaling with efferocytotic receptors or cellular metabolic modulators can restore defective clearance and quell autoimmune inflammation by resetting macrophage programs.

Naturally-derived nanomedicine, such as EVs with immunomodulatory effects, can be leveraged to re-educate macrophages and restore their efferocytotic capacity. For example, mesenchymal stem cell (MSC)-derived EVs have been used for treating lupus models, and intravenous infusion of MSC-EVs promoted an anti-inflammatory phenotype in recipient macrophages and enhanced their efferocytosis of ACs. As a result, lupus mice with MSC-EV therapy had reduced renal inflammation, increased clearance of nuclear debris, and corrected T-cell responses (reduced Th17 cells while expanded Treg cells) 216. Furthermore, impaired extracellular nuclease activity (e.g., DNASE1L3 loss-of-function) permits the accumulation of apoptotic cell microparticle-associated chromatin DNA and promotes anti-dsDNA/anti-chromatin autoimmunity and lupus-like disease 217. Accordingly, a promising therapeutic approach involves nanoparticle-mediated delivery of DNase to correct this enzymatic deficiency and enhance nucleic acid degradation. These reports illustrate a potent conceptual shift in treating autoimmune diseases: correction of intrinsic efferocytetic defects rather than broadly suppressing the immune system. The combinatorial modularity also showcases how nanotherapeutics can be tailored and layered to meet the complex immunological challenges of autoimmune diseases.

### 4.3. Neurodegenerative diseases

In neurodegenerative disorders, such as Alzheimer's and Parkinson's disease, inefficient clearance of apoptotic neurons and protein aggregates can amplify neuroinflammation and tissue injury. Microglia are the brain's resident macrophages and the principal efferocytes, making them a logical target for nanotherapeutics that aim to restore clearance functions while tempering inflammatory tone 43. A synthetic efferocytic receptor (SER), fusing an amyloid-β-binding single-chain variable fragment to a TIM4 extracellular scaffold and an ELMO1 intracellular signaling module, was designed to reprogram microglial engulfment. These SER-encoded mRNAs were delivered by mannosylated lipid NPs (MERLINs). After intrathecal administration, MERLINs penetrate meningeal and ventricular spaces and are preferentially taken up by microglia, thereby enhancing Aβ clearance and attenuating neuroinflammation in Alzheimer's disease models 218. Convergently, rejuvenating hypophagocytic, senescence-like microglia by silencing p16^Ink4a^ with PLGA NPs delivered via the cisterna magna yields preferential microglial uptake, robust p16 knockdown, increased lysosomal activity and Aβ phagocytosis, with improved spatial learning and memory in Alzheimer's disease model mice 219. These studies provide proof-of-concept that nanocarriers can deliver genetic instructions to reprogram microglial efferocytosis machinery in a disease-targeted manner.

Another therapeutic avenue is to use NPs that reprogramme how microglia handle toxic Aβ. For example, sugar-based amphiphilic macromolecule NPs (AM-NPs) that engage microglial scavenger receptors (SRA1, CD36, CD68) bind fibrillar Aβ, slow fibrillization and disrupt preformed fibers, dampen SR-mediated internalization, and enhance lysosomal degradation of Aβ, thereby reducing pro-inflammatory responses in microglia. This “eat smarter” paradigm illustrates how tuning substrate morphology and receptor usage can bias Aβ handling toward lysosomal clearance rather than pro-inflammatory signaling 220. Also, TFEB-dependent lysosomal competence is critical for microglial activation states in tauopathy/AD, positioning lysosomal biogenesis and functioning as tractable targets for efferocytosis-oriented nanotherapeutics 221.

In addition, nanotherapeutics aiming to attenuate oxidative stress that impairs efferocytosis in microglia have also been reported. For example, Ceria (CeO_2_) nanoclusters might act as catalytic ROS scavengers for lowering microglial ROS and preserving endolysosomal handling of Aβ in cellular models 222. While these results are promising, emerging solutions for overcoming the BBB barrier, such as RAGE-targeting systems, transferrin-receptor (TfR) “brain-shuttle” NPs/biologics, and nose-to-brain (intranasal) administration, may provide further therapeutic benefits 223-225. More combinatorial approaches are likely essential because neurodegeneration intertwines protein aggregation, cell death, and chronic neuroinflammation, with microglial efferocytosis sitting at the nexus of injury resolution 3. Safety concerns in the CNS require nanoplatforms with low immunogenicity and neuronal compatibility; accordingly, many efforts prioritize biocompatible carriers. Nanomedicines might help rebalance injury and repair in the brain, but rigorous work remains to secure precise human targeting and long-term safety.

### 4.4. Inflammatory disorders

Diverse forms of acute tissue injuries, such as acute lung injury (ALI), sepsis, acute kidney injury (AKI), myocardial infarction (MI), and intracerebral hemorrhage, are characterized by massive cell death, where timely efferocytosis is pivotal for returning inflamed tissues to homeostasis. To date, nanomedicines have been designed to promote resolution by boosting efferocytosis while tempering collateral inflammation 226. PS-displaying or apoptotic membrane-coated nanocarriers preferentially engage lung macrophages and promote pro-resolving efferocytosis in ALI models. For example, an inhalable apoptotic membrane-coated antioxidant “nanozyme” (AOzyme@ACM) enhanced “eat-me” signaling and reduced alveolar inflammation in mouse ALI models 121. Likewise, a “two-step” strategy, neutrophil-targeted apoptosis-restoring NPs followed by macrophage-targeted efferocytosis-restoring NPs, has also mitigated severe ARDS in preclinical models 227. Sepsis involves systemic inflammation and often immunosuppression, while the MFGE8-containing exosomes derived from immature dendritic cells (IDC-exosomes) could augment macrophage efferocytosis and improve survival in rodent sepsis, suggesting that “bridge-molecule” augmentation may be a potent therapeutic target in this state 228**.** Multiple types of acute organ injuries commonly couple oxidative stress and insufficient corpse clearance with reparative failure, which may be resolved by nanomedicines PS-presenting liposomes reprogrammed cardiac macrophages toward reparative phenotypes and improved postinfarct remodeling in MI models 65. In AKI models, KIM-1 (a kidney injury protein)-targeted black phosphorus nanoplatform loaded with 4-octyl-itaconate (4-OI) accumulated in injured kidneys, which scavenged ROS and restored pathways linked to efferocytosis 229. Neutrophil-like and pH-responsive pro-efferocytic NPs improved erythrophagocytosis and neurological recovery in mice with intracerebral hemorrhage 113.

Natural nanomedicines with efferocytosis-modulating effects have also been used. GAS6-enriched MSC-EVs enhanced efferocytosis in an ischemia/reperfusion-induced acute liver injury model 67; some of them might be edging toward the clinic. Infusion of early apoptotic cells (Allocetra™-OTS) for sepsis/cytokine storm syndromes 230, while apoptotic extracellular vesicles (ApoEVs) represent a cell therapy-like modality with advantages in scalable manufacturing and controlled delivery compared with whole apoptotic cell infusion 231. The multifunctional potential of nanoplatforms is compelling, as they can deliver payloads and actively engage macrophages to boost efferocytosis. Key challenges are safety and timing; mis-timed interventions can blunt necessary host responses, while well-timed therapies can facilitate resolution, underscoring the need for precise therapeutic windows. Nanomedicines with targeted delivery and controlled release effects are well suited to provide the required spatiotemporal control for such indications.

### 4.5. Cancers

Tumors coopt efferocytosis as an immune evasion mechanism. Many cancer cells overexpress “don't eat me” signals (notably CD47) to avoid phagocytosis, while the efferocytosis of apoptotic cancer cells by TAMs induces an immunosuppressive M2-like phenotype that dampens antitumor immunity. For cancer therapy, nanomedicines therefore pursue two complementary goals: enhancing the phagocytosis of tumor cells by TAMs and inhibiting the anti-inflammatory program initiated after the phagocytosis that causes immunosuppression. A prominent strategy is NP-mediated phagocytosis checkpoint blockade. For example, a degradable mesoporous silica NP co-delivering an anti-CD47 antibody and doxorubicin provided the two signals required for efficient engulfment—CD47 blockade to disable the inhibitory cue and doxorubicin-induced immunogenic cell death to expose calreticulin as an “eat-me” signal, thereby promoting macrophage phagocytosis and strengthening T-cell responses in murine tumor models 115. Similarly, a mannose-decorated liposome that co-delivers the TLR7/8 agonist resiquimod (R848) and a high-affinity SIRPα decoy protein (CV1) drives uptake by M2-like TAMs, repolarizes them toward M1, blocks the CD47-SIRPα axis, and enhances phagocytosis; in the MC38 model, this nanoformulation markedly reduced tumor burden, increased cytotoxic T-cell infiltration, and—upon systemic administration—showed no detectable hematologic or histopathologic toxicity 232.

Beyond antibody formulations, researchers have cloaked NPs with genetically engineered cell membranes that display high-affinity SIRPα variants, enabling *in situ* binding to tumor cell CD47, local checkpoint blockade, and restoration of macrophage phagocytosis 233. In addition to CD47, nanotherapeutics are being created to target alternative phagocytosis checkpoints such as CD24-Siglec-10, which certain cancers exploit to suppress macrophages 142. A recent study conjugated an anti-CD24 monoclonal antibody to the surface of biodegradable nanospheres, which degraded cell membrane CD24, blocked CD24-Siglec-10 signaling, and augmented macrophage phagocytosis 117.

Another vital strategy is the suppression of phagocytic receptors on tumor-associated phagocytes to prevent the clearance of apoptotic tumor cells and their subsequent transition into an immunosuppressive state. The TAM receptor family (Tyro3, Axl, MerTK) is a prime target in this regard. A glycopolymer-based NP was developed to deliver a small-molecule MerTK inhibitor (UNC2025) to TAMs in the TME. These NPs accumulated in CD206^+^ TAMs and inhibited efferocytosis of apoptotic tumor cells, thereby preventing TAMs from entering an anti-inflammatory state 129. In addition to blocking suppressive pathways, there is an interest in delivering “find-me” signals to tumors to recruit immune cells. The ATP-conjugated PLGA NPs could present ATP into tumors, thereby recruiting antigen-presenting cells while co-delivering paclitaxel to induce immunogenic cell death; combination with anti-PD-1 achieved complete regression in the CT26 colorectal tumor model and established immune memory 107, suggesting that efferocytosis-modulating NPs can synergize with other treatments, such as chemicals and anti-PD1 therapy. In the future, nanotherapeutics are proving to be the precise tools required to execute these nuanced interventions, holding promise for more effective and personalized cancer immunotherapy.

Collectively, current studies highlight unifying principles by which nanomedicines modulate efferocytosis for therapeutic gain: the ability to achieve precision targeting and controlled payload release, which minimizes off-target effects and enhances the therapeutic index. Common design elements include surface functionalization with ligands (e.g., phosphatidylserine mimics or macrophage-specific peptides) to facilitate phagocyte uptake, biomimetic properties that recapitulate apoptotic cell cues, and microenvironment-gated activation controls (e.g., pH- or ROS-sensitive release). While these shared mechanisms provide a foundational framework, disease-tailored designs remain essential because defective efferocytosis, tissue microenvironments and therapeutic goals differ substantially across indications. For instance, in atherosclerosis, nanomedicines aim to inhibit "don't-eat-me" signals (e.g., CD47) while boosting receptor activity (e.g., MerTK) and repairing lipid-handling programs needed for repeated rounds of efferocytosis; in autoimmune disease, nanomedicines often employ apoptotic mimicry to reimpose tolerogenic macrophage programs in a controlled manner. Cancer most clearly illustrates the need for mechanism-driven directionality: nanomedicines can enhance tumor cell phagocytosis via checkpoint blockade, yet they also inhibit TAM efferocytosis of apoptotic tumor cells and its downstream immunosuppressive program to restore antitumor immunity. Accordingly, whether to enhance or inhibit efferocytosis should be decided by the dominant pathogenic mechanism: enhancement is generally favored when the accumulation of apoptotic cells is a proximal driver of pathology (e.g., secondary necrosis, autoantigen exposure, and necrotic core formation), whereas inhibition may be rational when its downstream program is predicted to therapy resistance, most prominently in cancer. These considerations underscore that the translational challenge lies in precision—identifying the right compartment, phagocyte subset and disease stage for enhancement versus inhibition, while implementing spatiotemporal control to maximize benefits and minimize systemic liabilities.

## 5. Progress of efferocytosis-modulating therapies in clinical trials

Although the results from preclinical studies are promising, more important, their therapeutic potential requires further verification in clinical trials. Several translational strands have suggested that macrophage-engaging nanotherapies are approaching first-in-human evaluation, while closely related clinical advances, particularly localized or tumor-selective blockade of the CD47-SIRPα checkpoint, are already underway. For example, intratumoral delivery of the SIRPα-Fc decoy TTI-621 has shown activity in cutaneous T-cell lymphoma in a phase 1 setting (NCT02890368), and a protease-activated (masked) anti-CD47 antibody, SGN-CD47M, is being tested in a phase 1 trial in solid tumors (NCT03957096). In parallel, localized delivery of mRNAs encoding CD47 inhibitors has been reported preclinically as another route to confine target engagement 234, 235. However, until now, the overall clinical results have remained preliminary **(Table 4)**. For example, while intralesional TTI-621 demonstrated signs of activity in CTCL, definitive benefits have not been established, and the SGN-CD47M trial was terminated without reported efficacy (officially attributed to portfolio prioritization), indicating that these approaches require further assessment. These early experiences underscore a central design priority for next-generation efferocytosis-modulating nanomedicines: mitigating systemic toxicity through spatiotemporal control of target engagement and pharmacological activity. For instance, protease-sensitive nanosystems can carry “masked” payloads that remain inert during circulation and then release active drugs in enzyme-rich tumor sites, analogous to probody therapeutics, thereby sparing healthy cells 236, 237.

For the treatment of inflammatory disorders, specialized pro-resolving mediators (SPMs), including resolvins, protectins, maresins, and lipoxins, have advanced into early clinical evaluation (e.g., OMEGA-SPM-DOSE, NCT02719665), supporting the feasibility of resolution-directed interventions relevant to efferocytosis biology 238. At the same time, prior regulatory successes, from liposomal doxorubicin (Doxil) to lipid-NP mRNA vaccines, demonstrate that NP platforms can achieve clinical and manufacturing acceptance when supported by robust CMC and safety packages 239, 240** (Table 4)**. Looking ahead, it is plausible that the first trials explicitly branded as “pro-efferocytic NPs” or “macrophage-checkpoint-inhibitor NPs” will emerge initially in oncology, with cardiovascular indications to follow contingent on safety, as suggested by large-animal studies in atherosclerosis that reduced vascular inflammation via pro-efferocytic nanotherapeutics 9.

The preclinical success across diverse disease models provides compelling evidence that targeted modulation of efferocytosis via nanotherapeutics can prevent and even reverse pathology. From the chronic inflammation of atherosclerosis to the fulminant cytokine storm of sepsis, efferocytosis-modualting nanomedicines are proving remarkably versatile in tilting the balance toward disease resolution. A recurrent theme in these examples is combinatorial design: integrating multiple mechanisms (targeting motifs, payloads and biomimetic surface cues) in one nanosystem to address the multifaceted nature of efferocytosis dysfunction. This modular combinability is a distinctive strength of nanotherapeutics and underpins strategies that co-deliver immune-modulating signals with disease-specific targeting. Equally important is transferability: the conceptual modules (PS coating, MerTK agonist, CD47 blocker, etc.) are, in many cases, interchangeable across diseases. For example, a PS-coated NP that works in lung injury might be adapted to target kidney inflammation, or a neutrophil-membrane vesicle used in stroke could be repurposed for myocardial infarction — the principles of efferocytosis are conserved, even if the context differs. This bodes well for accelerating development, as success in one domain can inform another. Additionally, the next steps will involve confirming these benefits in large animal models and early human trials.

## 6. Challenges and future perspectives

To date, advancements in this field are impressive, but some challenges and key problems still exist and need to be resolved. For example, targeted nanocarrier-mediated drug delivery helps confine phagocyte activity, but perfect specificity remains elusive. Liver Kupffer cells avidly sequester many formulations, and the amounts of drugs delivered to target tissues (e.g., solid tumors) are relatively small (~0.7% of the injected dose) 241, 242. Moreover, marker overlap between target cells further complicates selectivity. For example, while CD206 (mannose receptor) is widely used to target M2-like TAMs, it is also expressed on Kupffer cells and some DCs or sinusoidal endothelial cells, and healthy tissue macrophages can be unintentionally engaged 243, 244. A rational solution is “logic-gated” targeting that layers cell-type ligands with disease-site triggers (e.g., pH, ROS, enzymes) and controlled-release kinetics to achieve selective activation only where and when desired 245, 246.

Beyond targeting, achieving precise spatiotemporal control of efferocytosis modulation is critical because hyperactivated efferocytosis can suppress needed early inflammatory defenses against infection or tumors, whereas excessive or prolonged inhibition risks autoimmunity or non-resolving inflammation 247. Thus, temporal and spatial control is required for nanomedicines, which might be achieved with stimuli-responsive or logic-gated nanomaterials that release payloads only in disease microenvironments. In some diseases (e.g., sepsis and lung infections), early enhancement of pro-resolving programs via efferocytosis can blunt antimicrobial defenses and worsen outcomes, whereas later delivery aids resolution and repair 52, 247. This timing-dependent paradox might be resolved using a “two-phase” nanomedicine: a first phase to augment efferocytosis, followed by an adjuvant phase to ensure antigen presentation. Furthermore, many efferocytosis nodes are pleiotropic, and MerTK signaling and TGF-β not only enforce immune quiescence but can also drive profibrotic remodeling when chronically engaged 248. Thus, anticipating and managing these pleiotropic outcomes will be part of the development process, engineering safety mechanisms such as limited exposure windows, using modules that self-destruct or release an inhibitor after a period, may all be part of future designs.

Immunogenicity and long-term safety remain major translational barriers for efferocytosis-targeting nanomedicines because engineered particles can be sensed as foreign and elicit innate and/or adaptive immune responses 249, 250. Systemically administered nanoparticles may activate the complement cascade, which can reshape opsonization and phagocytic clearance and can also precipitate infusion reactions known as complement activation-related pseudoallergy (CARPA) 251. Upon repeat dosing, carrier-directed antibodies can drive the accelerated blood clearance (ABC) phenomenon; a canonical example is PEGylated liposomes, where a priming dose can induce anti-PEG IgM that binds subsequent doses and promotes complement activation, thereby eroding the “stealth” benefit of PEG 252. These complement-antibody interactions can reduce exposure and efficacy and may also increase acute safety liabilities, underscoring the need to prospectively evaluate and mitigate CARPA/infusion-reaction risk during clinical translation. Over longer time horizons, the intracellular fate of nanomaterials in professional phagocytes is pivotal: biopersistent particles can accumulate in lysosomes and disrupt autophagy-lysosome flux, contributing to “lysosomal nanotoxicity” phenotypes that may compromise phagocyte function 253. Consequently, first-in-human development should prioritize immunocompatible, biodegradable materials, optimize dose and dosing frequency, and integrate repeat-dose and large-animal testing that is sensitive to anti-PEG/anti-carrier antibodies and complement-mediated reactions, thereby reducing ABC and CARPA risk.

Efferocytosis-modulating nanomedicine can also be combined with, rather than replace, current standards of care. In cancer, for example, preclinical studies have shown that inhibiting MerTK-mediated efferocytosis or blocking the CD47 checkpoint enhances responsiveness to PD-1/PD-L1 blockade 72. In atherosclerosis, the clinically used drug statins may enhance the "eat-me" signals on macrophages 254, suggesting a potent synergistic interplay with nanotherapeutics that target efferocytosis. Thus, it is possible that efferocytosis-targeted nanomedicine will allow lower doses or shorter durations of some standard therapies (e.g., steroids) along with reduced side effects. Clinical trial designs will have to carefully incorporate these therapies with standard care, likely starting as an add-on in refractory cases, then moving to earlier lines if proven safe and beneficial. Regulatory considerations are also important, demonstrating that adding nanotherapeutics does not interfere with the pharmacokinetics or safety of standard drugs. Ultimately, the goal is to weave efferocytosis modulators into the fabric of existing treatment protocols, possibly as an induction therapy (induce remission in autoimmunity then maintain with conventional drugs) or as a maintenance add-on (keep giving periodically to prevent flares or plaque progression).

Achieving maximal clinical impact with these therapies will require robust biomarkers and imaging tools to monitor *in vivo* modulation of efferocytosis and inflammation resolution programs. To date, there is still no widely adopted, standardized clinical biomarker that has been rigorously validated to specifically track efferocytosis dynamics, reflecting the multi-step and context-dependent complexity of this process. Recent work has identified candidate indicators that could be leveraged as surrogate readouts, including soluble TAM receptors generated by proteolytic shedding (sTyro3, sAxl, and soluble Mer/sMerTK), which are measurable in blood or other biofluids and have been linked to dysregulated apoptotic cell clearance 255. In atherosclerosis, myeloid expression of a cleavage-resistant MerTK variant lowers soluble Mer (sol-Mer) and improves lesional efferocytosis 40. Similarly, in systemic lupus erythematosus, plasma sMer is increased during active disease and has been reported to decrease in parallel with improvements in disease activity (SLEDAI) after therapy 255. These findings support soluble TAM receptors as candidate surrogate markers of efferocytosis dysregulation and therapeutic response, but clinical deployment will require standardized assays, longitudinal validation across cohorts and careful adjustment for inflammation-related confounding factors.

Beyond soluble mediators, transcriptomic efferocytosis-related gene signatures are being explored as candidate biomarkers. In oncology, studies integrating bulk RNA-seq with single-cell analyses and machine-learning feature selection have derived efferocytosis-related gene sets associated with prognosis and with differences in immune infiltration and predicted immunotherapy response 256; in ulcerative colitis, multiomics integration identified efferocytosis-related hub genes whose baseline expression patterns correlated with clinical response to ustekinumab and showed moderate discriminative performance 257. Overall, translating such panels into clinically actionable precision-medicine tools will require standardized assays, longitudinal sampling and validation against more direct measures of efferocytosis to establish both specificity and dynamic response ranges.

A parallel frontier in this field is the advancement of molecular imaging tools that can noninvasively quantify two crucial elements: the tissue burden of apoptotic cells and the biodistribution/lesional accumulation of nanomedicines at sites of disease. Radiolabeled Annexin A5/V (PS-binding) has been evaluated in clinical atherosclerosis studies 258. Early clinical and validation studies suggest that Annexin-based imaging can highlight plaques with higher cell-death burden and features considered more “vulnerable”; however, the signal primarily reports cell-death burden and therefore provides only indirect evidence of impaired efferocytosis 259. To more directly visualize efferocytosis, the pH-sensitive probe AnxA5-pHrodo developed by Stöhr and colleagues offers an elegant solution: it is dim at neutral pH but becomes brightly fluorescent once labeled apoptotic targets are internalized into acidic phagosomes/lysosomes, enabling flow cytometric and microscopic discrimination between surface-bound targets and truly engulfed cargo and thereby supporting quantitative assessment of efferocytic capacity 260.

Beyond imaging cell corpses, there is growing interest in noninvasively tracking the *in vivo* distribution and lesion-level deposition of therapeutic nanoparticles in real time. Theranostic nanomedicines can be equipped with imaging reporters, such as iron oxide/USPIO contrast for MRI, radiolabels for PET/SPECT, or fluorescent dyes for optical imaging, to quantify biodistribution and intralesional accumulation 261. Clinically, in a pilot study of patients with advanced solid tumors treated with nanoliposomal irinotecan (nal-IRI), higher ferumoxytol-MRI-derived lesion metrics at 1-24 hours were significantly associated with subsequent lesion shrinkage, supporting ferumoxytol MRI as a noninvasive predictor of heterogeneous nanoparticle deposition 262. Therefore, incorporating imaging readouts (e.g., MRI-traced nanoparticle deposition) into early-phase trials of efferocytosis-targeted nanomedicines could provide delivery/pharmacodynamic endpoints for patient-lesion stratification and early go/no-go decisions, thereby derisking larger outcome-focused studies.

One of the foremost translational challenges moving forward is to identify which patients will benefit most from efferocytosis-targeted therapies and to tailor interventions accordingly. Given the heterogeneity of efferocytosis across diseases and individuals, patient stratification based on molecular, cellular or disease-specific features is essential for maximizing efficacy. For example, patients with tumors highly overexpressing CD47 or having an abundance of CD47^+ tumor-derived extracellular vesicles in circulation may experience greater benefit from anti-CD47 agents. This is analogous to how HER2-positive breast cancer patients are selected for anti-HER2 therapy 263. Likewise, in atherosclerosis, patients with high CD47 expression in plaques or with elevated soluble MerTK in blood might be prime candidates for an efferocytosis-enhancing nanotherapy. Stratification can also draw on gene signatures; as noted, at the transcriptomic level, efferocytosis-related gene panels have been proposed across indications and can be associated with prognosis and therapy response. If a tumor's RNA sequencing reveals an efferocytosis gene signature associated with immunosuppressive macrophages, the patient might benefit from an efferocytosis-inhibiting approach (for example, MerTK blockade).

Imaging-based companion diagnostics are particularly attractive for nanomedicines because delivery varies substantially across patients and lesions; clinical and translational studies have used ferumoxytol-MRI to estimate lesion uptake/permeability and to predict deposition and response to therapeutic nanoliposomal drugs 262. This concept was highlighted by Cooley *et al.*, who argued that pre-treatment imaging of nanoparticle delivery could serve as a stratification tool to improve trial success rates for nanomedicines 264. In addition, novel PET tracers for apoptotic cells could identify patients with high apoptotic burden and low clearance; for example, an 18F-annexin PET scan might reveal which atherosclerosis patients have large areas of uncleared apoptotic cells in plaques, and these patients may be selected for pro-efferocytic therapy. Ultimately, a combination of biomarkers might be used to construct an “efferocytosis score” for patients, guiding enrollment in trials and individualized therapy plans. It should be noted that rigorous validation of these candidate biomarkers is needed; many have shown correlations in retrospective studies, but their predictive value in interventional settings remains to be proven.

Finally, there are significant scaled manufacturing and regulatory challenges that must be addressed to translate efferocytosis nanomedicines from bench to bedside. Nanomedicines typically present higher manufacturing complexity than small molecules or traditional biologics; achieving batch-to-batch consistency in sizes, surface attributes, and payloads under GMP is a well-recognized chemistry, manufacturing, and control (CMC) challenge (FDA-2017-D0759). Nanoplatforms often involve multiple components, each adding to cost and variability. This could affect commercial viability, especially if the target patient population is large (such as tens of millions with atherosclerosis). Future research needs to emphasize simplifying nanomedicine designs where possible or leveraging biomaterials that are easier to produce. Regulatory expectations are tightly coupled to manufacturing challenges: for complex nanoparticles, demonstrating robust CMC requires validated analytical methods to define and control critical quality attributes (CQAs)—including size distribution, morphology, surface properties/functionalization (e.g., ligand density, orientation and conformational state for actively targeted systems), and cargo loading/release, to support batch-to-batch consistency 265. Fortunately, the field is actively advancing such methods, including single-particle tracking and advanced spectroscopy, to strengthen nanoparticle quality control. Additionally, modular manufacturing approaches offer promising solutions; for example, scalable "generic" liposomes or lipid nanoparticles (LNPs) can be produced first, followed by the addition of disease-specific ligands through post-insertion or late-stage conjugation steps to standardize the majority of the process 266. Encouragingly, the mRNA-LNP experience during COVID-19 indicates that, with focused investment and demand, NP platforms can be scaled from lab to billions of doses. In the future, platformized and modular manufacturing processes will be pivotal to making efferocytosis-targeted nanotherapeutics commercially and clinically viable.

In summary, nanomedicine-based strategies for modulating efferocytosis have advanced considerably in recent years, and addressing the aforementioned challenges remains essential. In the future, interdisciplinary collaboration that integrates insights from immunology, materials science, and clinical medicine would foster innovative solutions. The field might be shifting from questions of feasibility (could this work?) to developments of nanoplatforms with advanced efficacy and biosafety (how do we make it work better and safer?). Ongoing research is poised to yield more refined targeting ligands and intelligent responsive nanoplatforms and to provide deeper insights into efferocytosis biology for therapeutic exploitation. Ultimately, modulating the body's natural ability to handle apoptotic cells will emerge as a mainstream therapeutic approach, enabled primarily by the precision and customizability of nanotherapeutics.

## Supplementary Material

Supplementary table.

## Figures and Tables

**Table 1:** Evidence of dysregulated efferocytosis in diverse diseases

Diseases		Disregulated efferocytotic pathways	Pathological consequences	Model/Evidence level	Reference
**Autoimmune Diseases**	**Systemic Lupus Erythematosus (SLE))**	Defects in C1q/C3 opsonins; TAM receptors (MerTK, Axl, Tyro3); MFG-E8; SCARF1; TAM receptors.	Accumulation of apoptotic debris, release of nuclear autoantigens, autoantibody production, immune-complex deposition; lupus nephritis and multi-organ damage.	Human biopsy/serum; Mice, *in vivo* (lupus-prone models); macrophage & dendritic cell, *in vitro*	33, 34, 60, 61
	**Rheumatoid Arthritis (RA)**	ADAM10/17; MerTK; MFG-E8; dysregulated macrophage phenotypes.	Persistent synovitis and pannus; defective apoptotic cell clearance in joints, chronic inflammation and cartilage/bone erosion.	Human synovium/serum; Mice, *in vivo* (CIA/K/BxN arthritis); macrophage & synoviocyte, *in vitro*	36, 62
	**Inflammatory Bowel Disease (IBD)**	RUBCN/LAP axis; MerTK; MFG-E8.	Impaired clearance of apoptotic cells in the intestinal milieu; barrier disruption, chronic colitis, dysbiosis-associated inflammation.	Human intestinal biopsy/serum; Mice, *in vivo* (DSS/TNBS/IL-10^-/-^ colitis); macrophage & dendritic cell, *in vitro*	63
**Atherosclerosis**		ADAM10/17; MerTK; LXR/PPAR-ABCA1/ABCG1 lipid-efflux axis; CD47-SIRPα.	Defective clearance of apoptotic foam cells, growth of lipid-rich necrotic core, fibrous cap thinning, plaque destabilization and thrombosis.	Human plaque; Mice, *in vivo* (ApoE^-/-^/Ldlr^-/-^ models); foam cell, *in vitro*	7, 38, 64
**Neurodegeneration Diseases**	**Alzheimer's Disease (AD)**	TREM2-TYROBP signaling; APOE; TAM receptors; LAP.	Impaired clearance of apoptotic neurons and amyloid-β/tau aggregates; chronic neuroinflammation and synapse loss.	Human postmortem/genetics; Mice, *in vivo* (APP/PS1, 5xFAD); microglia, *in vitro*	43
	**Parkinson's Disease (PD)**	TREM2/TYROBP signaling; TAM receptors; altered debris handling.	Defective removal of α-synuclein aggregates and apoptotic dopaminergic neurons; persistent neuroinflammation;	Human postmortem/genetics; Mice, *in vivo* (MPTP-induced PD); microglia & astrocyte coculture, *in vitro*	45-47
**Inflammatory disorders**	**ARDS/ALI**	Neutrophil extracellular traps (NETs) and DNASE1/1L3 degradation pathways; MerTK; MFG-E8; C5a-C5aR1; cytokine storm.	Delayed clearance of apoptotic cells and NETs, sustained hyperinflammation, alveolar injury, respiratory failure.	Human ICU cohorts/BALF; Mice, *in vivo* (LPS/acid/ventilation-induced ALI); neutrophil & macrophage, *in vitro*	48
	**Sepsis**	NET overload and defective nucleases; (C5a-C5aR1); impaired MerTK; MFG-E8.	Inefficient corpse/NET clearance, immune paralysis, organ dysfunction.	Human ICU cohorts/blood; Mice, *in vivo* (CLP/LPS sepsis); neutrophil & macrophage, *in vitro*	49
	**Acute organ injury**	Overwhelming apoptotic burden with insufficient MerTK; MFG-E8; dysregulated resolution programs.	Accumulation of cell debris, persistent inflammation.	Human biopsy/serum; Mice, *in vivo* (ischemia-reperfusion/MI/AKI); macrophage, *in vitro*	65-67
**Cancers**		CD47-SIRPα; CD24-Siglec-10; TAM receptors (MerTK/Axl); IL-10, TGF-β; tumor-associated macrophage (TAM) phenotypes	Immune evasion and T-cell suppression, M2-like TAM polarization, immunosuppressive tumor microenvironment, angiogenesis and metastasis.	Human tumor datasets/biopsies; early-phase clinical studies targeting CD47/SIRPα and MerTK/Axl; Mice, *in vivo* (syngeneic/xenograft and spontaneous tumor models); macrophage, *in vitro*.	21, 59

**Table 2:** Conventional strategies for modulating efferocytosis

Strategy Category	Target pathways		Mechanisms of action	Model/Evidence level	Limitations		Reference
Small molecule Drugs	LXRα/β (NR1H3/NR1H2)		Promote lipid efflux (ABCA1/ABCG1↑) Transactivate MerTK Reprogram macrophages toward a pro-resolving state	Clinical (Phase I); Mice, *in vivo* (atherosclerosis); macrophage & dendritic cell, *in vitro*	Systemic LXR agonists can cause hypertriglyceridemia/steatosis; pathway crosstalk with TLR can suppress LXR targets during infection; cell-type and context dependence.		24, 68, 69
	PPARγ (e.g., pioglitazone)		Reprogram macrophages toward a pro-resolving state Cytoskeletal regulators (e.g. Rac1/Vav1 axis) Anti-inflammatory cytokines (IL-10) enhanced	Mice, *in vivo* (lung injury/MS models); macrophage & dendritic cell, *in vitro*	Metabolic side effects (weight gain, edema); variable efficacy across tissues; off-target nuclear receptor pleiotropy.		70
	ALX/FPR2 (e.g., columbamine as a biased agonist)		Enhances LC3-associated efferocytosis (LAP)	Mice, *in vivo* (colitis models); macrophage & dendritic cell, *in vitro*	Selectivity and bias of agonists; receptor expression varies across tissues; translational evidence still emerging.		71
	MerTK inhibitors (UNC2250, BMS-794833, etc.)		Pharmacologic MerTK inhibition generally suppresses efferocytosis Boost antitumor immunity by preventing clearance of apoptotic cells	Clinical (Phase I/II); Mice, *in vivo* (cancer models); macrophage & dendritic cell, *in vitro*	Impaired tissue resolution; potential toxicity if used outside oncology; off-target TAM inhibition (Axl/Tyro3).		72-75
Cytokines and Biologics	CD47-SIRPα axis (anti-CD47 mAbs; SIRPα-Fc fusion proteins)		Blocks the 'don't-eat-me' signal to enable macrophage engulfment of apoptotic/damaged cells and tumor cells	Clinical (Phase I/II/III) Mice, *in vivo* (cancer/atherosclerosis); macrophage & dendritic cell, *in vitro*	On-target anemia/thrombocytopenia; dosing/titration challenges (RBC sink); mixed Phase 3 outcomes and clinical holds in some indications.		76, 77
	Recombinant MFG-E8		Bridges phosphatidylserine on apoptotic cells to integrins (αvβ3/αvβ5) on phagocytes to augment recognition and internalization	Mice, *in vivo* (sepsis/ischemia/reperfusion); macrophage & dendritic cell, *in vitro*	Short half-life; context-dependent efficacy; manufacturing and delivery considerations.		44, 78
	IL-10/IL-4/IL-13		Pro-resolving cytokines that upregulate engulfment machinery (e.g. IL-10→Vav1→Rac1); Promote Macrophage alternative activation; Enhance clearance during resolution.	Clinical (selected biologics in development/approved, target-related); Mice, *in vivo* (atherosclerosis/ALI); macrophage & dendritic cell, *in vitro*	Systemic immunosuppression; dosing window for 'helpful vs harmful' responses; pleiotropy across tissues.		28, 79, 80
**Gene Therapy**	MERTK replacement in RPE (retinal dystrophy)	Gene replacement restores RPE efferocytosis of photoreceptor outer segments; Preserves photoreceptors in models of MERTK-associated retinopathy.		Mice, *in vivo* (RCS rat); macrophage & dendritic cell, *in vitro*	Vector delivery, durability and immunogenicity; indication-specific (ocular) rather than systemic.	81	
	Engineered efferocytic receptors (CHEF; e.g., TIM4-ELMO1 fusions)	Synthetic receptors couple PtdSer recognition to ELMO1-Rac1 signaling; Selectively boosting apoptotic-cell uptake.		Mice, *in vivo* (colitis/arthritis); macrophage & dendritic cell, *in vitro*	Cell engineering complexity; safety controls for over-phagocytosis; translational readiness.	82	
	CD47/SIRPα (e.g., CRISPR knockout)	Remove the CD47-SIRPα checkpoint to increase efferocytosis.		Mice, *in vivo* (cancer models) macrophage & tumor co-culture, *in vitro*	High editing efficiency needed; *in vivo* editing safety (on/off-target effects); delivery barriers; tumor heterogeneity	83-85	
	Macrophage ACSL1 inhibition; adoptive transfer of metabolically reprogrammed macrophages	Dampens NF-κB-driven inflammation; Shifts macrophages toward a pro-resolving state.		Mice, *in vivo* (atherosclerosis/diabetes); macrophage *in vitro*	*Ex vivo* manipulation and scalability; durability after transfer; disease specificity.	86	
							

**Table 3:** Nanomedicine-based strategies for modulating efferocytosis

Strategy Category	Applied nanomedicines	Target pathways	Formulations	Target cells	Biological outcomes	Reference
**Regulation of “find-me” signals**	PTX@NP_pD_-ATP	ATP/UTP, S1P, CX3CL1, LPC; dendritic-cell recruitment; antitumor immunity	Material: PLGA; Size: ~190 nm; Decorated: ATP Cargo: paclitaxel (PTX)	Dendritic cells; Macrophages	Enhance phagocyte recruitment to disease sites	107
	S1P-PS-MMV@SPD	S1P (find-me); phosphatidylserine (eat-me)	Material: Macrophage-membrane nanovesicles (MMV); Size: ∼416.4 ± 1.9 nm; Decorated: S1P and PS Cargo: spermidine	Macrophages	Promote macrophage recruitment and uptake	108
**Regulation of “eat-me” signals**	PSNP@DEM	Phosphatidylserine (PS); anti-inflammatory efferocytic signaling	Material:PLGA; Size:150-200 nm; Decorated: PS Cargo: dexamethasone	Alveolar/lung macrophages	Increase macrophage uptake; reduce inflammation; alleviate lung injury	105
	Engineered neutrophil apoptotic bodies (eNAB)	Phosphatidylserine and other native eat-me signals	Material: Neutrophil apoptotic-body membrane cloaked mesoporous silica nanoparticles; Size: ~300 nm; Decorated: Neutrophil apoptotic-body membrane	Macrophages	Enable rapid binding/ingestion; reprogram toward pro-resolving phenotype	109
	mBM-MSC-EV	GAS6; MerTK signaling	Material: Extracellular vesicles derived from MSC; Size: 100-200 nm; Cargo: enrichment of GAS6	Hepatic macrophages	Accelerate apoptotic-cell clearance; dampen inflammation	67
	Bispecific tumor-transforming nanoparticles (BiTNs)	Eat-me ligands; tumor-binding moieties	Material: PEG-PLGA; Size: ~100 nm; Decorated: conjugated with anti-HER2 and SLAMF7	Macrophages; Tumor cells	Physically bridge targets to induce efferocytosis/phagocytosis	110
**Regulation of phagocytic receptors**	MerTK-displaying hybrid-membrane nanovesicles (HMNVs)	MerTK receptor	Material: Hybrid membranes from MerTK-overexpressing macrophages; Size: 150-200 nm; Decorated: MerTK	Lesional macrophages	Donate functional MerTK; enhance local efferocytosis	111
	PS-lipos-AuNC@LXR agonist	LXR; MerTK expression	Material: silver nanocubes and liposomes; Size: 100-150 nm; Decorated: PS Cargo: T0901317	Macrophages	Upregulate MerTK; promote apoptotic-cell uptake and anti-inflammatory programs	112
	D&G@NPEOz	ADAM17 (MerTK shedding); LXR	Material: pH-responsive neutrophil membrane-based nanoplatform (NPEOz); Size: 50 nm; Cargo:GW280264X (an ADAM17 inhibitor) and desmosterol (an LXR agonist)	Brain phagocytes (e.g., microglia)	Prevent MerTK shedding; enhance efferocytosis (e.g., erythrophagocytosis)	113
	Efferocytosis-Inhibiting Nano-BMS	MerTK	Material: PLGA and mPEG-PLGA; Size: ~106 nm; Cargo: MerTK inhibitor (BMS777607)	Tumor-associated macrophages (TAMs)	Block efferocytosis to limit tumor immune evasion/growth	114
**Regulation of “don't eat me” signals**	aCD47-DMSN	CD47-SIRPα axis; calreticulin ('eat-me') exposure	Material: mesoporous silica nanoparticle; Size: ~152 nm; Decorated: anti-CD47 antibodies; Cargo: doxorubicin	Tumor cells; Macrophages	Multivalent CD47 blockade; induce eat-me signaling; enhance phagocytosis	115
	Single-walled carbon nanotubes (SWNTs)	SIRPα-SHP1 signaling	Material: PEG-functionalized SWNTs; Size: diameter of 5-6 nm; Cargo: inhibitor of SHP-1	Plaque macrophages	Reactivate efferocytosis in plaques while minimizing anemia risk	41
	ZrMOF/C@P	CD47 checkpoint; STING pathway	Material: zirconium-based metal-organic framework and RAW264.7 cell membrane; Size: 190.6 nm; Decorated: Pep20, MMP2, and M2pep Cargo: STING agonists	Tumor cells; Macrophages	Block CD47; enhance phagocytosis with reduced hematologic toxicity	116
	Nano lysosome-targeting chimeras (LYTACs)	CD24-Siglec-10 axis	Material: LYTAC and *N*-acetylgalactosamine(GalNAc); Size: ~200 nm; Decorated: CD24 antibodies	Tumor cells; Macrophages	Disrupt CD24-Siglec-10; enhance macrophage phagocytosis	117
**Regulation of internalization and degradation**	Silica nanoparticles	Rac1 GTPase; actin polymerization	Material: Silica	Macrophages	Boost Rac1 activity; improve phagocytic internalization	118
	Engineered lysosome-acidifying nanoparticles	Lysosomal acidity; hydrolase activity	Material: poly(butylene tetrafluorosuccinate-co-succinate) polyesters; Size: ~100 nm	Macrophage lysosomes	Re-acidify lysosomes; recover degradative capacity	119
**Metabolic regulation for continual efferocytosis**	MMR-Lobe	PPARγ; ABCA1/ABCG1; cholesterol efflux	Material: thiolated glycol chitosan; Size: 300-350 nm; Cargo: lobeglitazone	Macrophages	Promote cholesterol efflux; anti-inflammatory gene programs; sustain efferocytosis	120
	AOzyme@ACM	ROS scavenging; mitochondrial integrity	Material: silica NPs core, Cu NPs and apoptotic cell membrane; Size: ~150 nm; Decorated: apoptotic cell membrane	Macrophages	Reduce oxidative stress; reactivate continual efferocytosis	121

**Table 4:** Clinical progress of efferocytosis-modulating approaches and representative clinically validated nanoparticle platforms

Trial registration	Phase	Drug/Nanoparticle Type	Primary Endpoint	Key Results	Current Limitations
NCT02890368	Phase 1	TTI-621 (SIRPα-Fc decoy fusion protein)	Safety/tolerability (AEs); activity signals (lesion response metrics)	Intralesional administration showed antitumor activity in CTCL, with ≥50% reduction in CAILS score in 34% of patients; well tolerated; treatment-related AEs mostly grade 1-2.	Definitive benefits not established; terminated early; limited to specific indications like mycosis fungoides; requires further assessment for broader efficacy and systemic use.
NCT03957096	Phase 1	SGN-CD47M (protease-activated masked anti-CD47 antibody)	Safety/tolerability; PK; immunogenicity; preliminary antitumor activity	Terminated (sponsor decision/portfolio prioritization); no efficacy dataset established publicly.	Attributed to portfolio prioritization; no efficacy reported; potential systemic toxicity concerns; requires further evaluation for safety and activity in solid tumors.
NCT02719665	Early-phase (pilot)	Specialized pro-resolving mediators (SPMs) enriched marine oil supplement	Changes in lipid mediators and leukocyte phenotype; safety.	Significant survival benefit in metastatic pancreatic cancer postgemcitabine; median OS 6.1 months for MM-398 + 5-FU/LV vs 4.2 months for 5-FU/LV alone; improved PFS and response rates.	Modest sample size; unblinded and short-term design; lack of clinically meaningful endpoints; preliminary evidence only; feasibility demonstrated but definitive benefits in inflammatory disorders not established.
NCT01494506	Phase 3	MM-398 (liposomal irinotecan) with or without 5-FU/LV	Overall survival (OS)	MM-398 with 5-FU/LV improved OS vs control (reported median OS benefit and favorable HR in final analyses).	Potential toxicities like diarrhea and neutropenia; limited to patients who failed prior gemcitabine; dose modifications may be needed.
NCT01960348	Phase 3	Patisiran (ALN-TTR02; lipid nanoparticle (LNP)-siRNA)	Change in modified Neuropathy Impairment Score +7 (mNIS+7) at 18 months.	Significant improvement in neuropathy scores; median change in mNIS+7: -6.0 points for patisiran vs +28.0 for placebo; overall survival and cardiac benefits observed; well-tolerated with infusion-related reactions.	Requires IV infusion; long-term follow-up in extension study; limited to hereditary transthyretin amyloidosis patients; no major limitations noted but ongoing monitoring for safety.
NCT04368728	Phase 3	BNT162b2 (LNP-mRNA vaccine)	Incidence of confirmed COVID-19 cases from 7 days after second dose; safety.	Vaccine efficacy of 95% against COVID-19; well-tolerated; immunogenicity confirmed in adolescents and adults.	requires ultracold storage; durability/boosting needs; variant immune escape; rare adverse events (e.g., myocarditis) require pharmacovigilance and risk-stratified use.
NCT04470427	Phase 3	mRNA-1273 (LNP-mRNA vaccine)	Number of participants with first occurrence of COVID-19 starting 14 days after second dose; safety.	Vaccine efficacy of 94.1% against COVID-19; well tolerated; serious adverse events low and comparable to placebo.	Similar class limitations: durability/boosting, variant drift, rare adverse events and ongoing postmarketing safety surveillance.

**Abbreviations:** AEs: adverse events; CAILS: Composite Assessment of Index Lesion Severity; CTCL: cutaneous T-cell lymphoma; OS: overall survival; PK: pharmacokinetics; SPMs: specialized pro-resolving mediators; 5-FU/LV: 5-fluorouracil/leucovorin.

## Abbreviations

ABCA1: ATP-binding cassette transporter A1
AC: apoptotic cell(s)
AD: Alzheimer's disease
ADAM17: a disintegrin and metalloproteinase 17
ApoE: apolipoprotein E
ATP: adenosine triphosphate
AXL: AXL receptor tyrosine kinase
BTB: blood-testis barrier
CD24: cluster of differentiation 24
CD47: cluster of differentiation 47
CX3CL1: C-X3-C motif chemokine ligand 1 (fractalkine)
IL-10: interleukin 10
LAP: LC3-associated phagocytosis
LPC: lysophosphatidylcholine
LXR: liver X receptor
MerTK: MER proto-oncogene tyrosine kinase
MFG-E8: milk fat globule-EGF factor 8 (lactadherin)
NP: nanoparticle(s)
PLGA: poly(lactic-co-glycolic acid)
PPAR: peroxisome proliferator-activated receptor
PS: phosphatidylserine
PtdSer: phosphatidylserine
Rac1: Ras-related C3 botulinum toxin substrate 1
ROS: reactive oxygen species
SIRPα: signal regulatory protein alpha
TAM: Tyro3-Axl-Mer (receptor family)
TFEB: transcription factor EB
TGF-β: transforming growth factor beta
TIM-4: T-cell immunoglobulin and mucin domain-containing protein 4
UTP: uridine triphosphate
