# Supplementary material for: Advancements in nanomedicine for the therapeutic regulation of efferocytosis: opportunities and challenges
Source: Theranostics. 2026 Mar 25;16(10):5537–70. doi: 10.7150/thno.128155 (PMC13080804; doi:10.7150/thno.128155)
Supplement: Supplementary file 1 — Supplementary table. [file thnov16p5537s1.pdf]

Advancements in nanomedicine for therapeutic regulation of efferocytosis: opportunities and challenges

Zehuan Lin[1], Xiyue Zhou[1], Shuyun Liu[1], Meihua Wan[2], Jingping Liu[1]*

[1] Department of General Surgery and NHC Key Laboratory of Transplant Engineering and Immunology, Frontiers Science Center for Disease-related Molecular Network, West China Hospital, Sichuan University, Chengdu 610041, China

[2] West China Center of Excellence for Pancreatitis, Institute of Integrated Traditional Chinese and Western Medicine, West China Hospital, Sichuan University, Chengdu 610041, China

*Corresponding Authors:

Jingping Liu (liujingping@scu.edu.cn)

Address: NHC Key Laboratory of Transplant Engineering and Immunology, West China Hospital, Sichuan University, No. 2222 Xinchuan Road, Chengdu 610041, China

Tel: +86-28-85164029, Fax: +86-28-85164030

Table S1. Therepeutic effects of nanomedicine in diverse diseases

Diseases		Applied nanomedicines	Mechanisms of action	Targeting & administration routes	Therapeutic outcomes	Reference
**Autoimmune disease**	**Systemic Lupus Erythematosus (SLE)**	PS-liposome-coated gold nanocages with LXR agonist	Enhance eat-me signaling (PS); upregulate MerTK via LXR	Administration not specified; SLE mice	Enhanced clearance; reduced autoantibodies; alleviated kidney damage	[121]
		Mesenchymal stromal cell-derived exosomes	Enhance efferocytosis; promote anti-inflammatory phenotype	Intravenous infusion; lupus mice	Alleviated disease; reduced renal inflammation; corrected T-cell response	[209]
	**Rheumatoid Arthritis (RA)**	Efferocytosis-informed nanoimitator (EINI) with siRNA and metformin	Activate PS signaling; reprogram macrophages via siIRF5	Heparin decoration for inflamed endothelium; RA mice	Reprogrammed macrophages; mitigated joint pathology	[73]

**Type 1 diabetes**	PS-liposomes with insulin peptide	Antigen-specific tolerization via PS signaling	Administration not specified; NOD mice	Shifted macrophage phenotype; increased Treg responses	[210]
**Atherosclerosis**	Hybrid-membrane nanovesicles (HMNVs) with MerTK	Enhance eat-me signaling via MerTK receptor delivery	Magnetic navigation; intravenous; ApoE$^{-/-}$ mice	Reduced plaque inflammation; improved efferocytosis	[120]
	sHDL nanoparticles carrying LXR agonist (T0901317)	Enhance eat-me signaling by upregulating MerTK (LXR activation)	Atheroma targeting; intravenous; ApoE$^{-/-}$ mice	Enhanced cholesterol efflux; inhibited lesion progression	[211]
	SHP-1 inhibitor–loaded single-walled carbon nanotubes (SWNTs)	Block don't-eat-me signaling (CD47/SIRPα axis) by inhibiting SHP1	Macrophage-specific delivery; intravenous; ApoE$^{-/-}$ mice and porcine model	Reduced necrotic core; no hematologic toxicity	[53]
	CpG-conjugated silver nanoparticles (CpG-AgNPs)	Macrophage reprogramming via	Route not specified; ApoE$^{-/-}$ mice	Enhanced efferocytosis; lesion control	[212]

		TLR9 agonism			
	HA-coated macrophage-membrane nanoparticle (HA-M@P@(AT+Met))	Macrophage reprogramming toward M2 polarization	Biomimetic coating; in vivo model not specified	Reduced necrotic cores; stabilized plaques	[213]
**Alzheimer’s Disease (AD)**	MERLINs (synthetic efferocytic receptor mRNA delivered in LNPs)	Reprogram microglia for amyloid-β clearance	Intrathecal delivery; mannosylation for microglia; AD models	Enhanced Aβ clearance; attenuated neuroinflammation	[214]
	PLGA nanoparticles silencing p16Ink4a	Rejuvenate microglia to restore phagocytic capacity	Intracisternal magna delivery; 5xFAD mice	Increased Aβ phagocytosis; reduced plaque burden; improved memory	[215]
	Sugar-based amphiphilic macromolecule nanoparticles (AM-NPs)	Reprogram microglial handling of Aβ; enhance lysosomal	Scavenger-receptor engagement; cellular and in vivo models	Accelerated lysosomal degradation; dampened inflammation	[216]

			processing			
**Inflammatory disorders**	**ARDS/ALI**	Inhalable apoptotic-membrane–coated antioxidant nanozyme (AOzyme@ACM)	Enhance eat-me recognition via apoptotic membrane; antioxidation	Inhalation; mouse ALI model	Reduced alveolar inflammation; improved outcomes	[130]
		Two-step nanoparticle strategy targeting neutrophils then macrophages	Restore neutrophil apoptosis and macrophage efferocytosis	Administration not specified; preclinical ARDS models	Mitigated severe ARDS	[217]
	**Sepsis**	MFG-E8–enriched exosomes	Augment bridging molecule availability to enhance efferocytosis	Rodent sepsis models	Improved survival; favorable inflammatory readouts	[218]
	**Acute organ injury**	PS-presenting liposomes	Reprogram macrophages toward a reparative	Post-MI delivery; myocardial infarction models	Improved post-infarct remodeling	[75]

		phenotype			
	KIM-1–targeted black-phosphorus loaded with 4-octyl itaconate (4-OI)	Restore efferocytosis pathways; scavenge reactive oxygen species	KIM-1 targeting; acute kidney injury models	Restored repair pathways	[219]
	GAS6-enriched MSC extracellular vesicles	Enhance efferocytosis via GAS6–MerTK axis	Liver injury models	Mitigated liver injury	[77]
	Neutrophil-like, pH-responsive nanoparticles	Improve erythrophagocytosis by brain phagocytes	Intracerebral hemorrhage models	Improved neurological recovery	[122]
**Cancer**	Anti-CD47 antibody and doxorubicin co-delivering mesoporous silica nanoparticle	Block don’t-eat-me signaling (CD47) and induce calreticulin eat-me exposure	Administration not specified; murine tumor models	Promoted phagocytosis; strengthened T-cell response	[124]

	Mannose-decorated liposome with R848 and SIRPα decoy (CV1)	Block don’t-eat-me signaling (CD47/SIRPα); repolarize TAMs	Mannose-receptor targeting; systemic in MC38 model	Reduced tumor burden; increased T-cell infiltration; no toxicity	[220]
	Anti-CD24 antibody–conjugated nanospheres	Block don’t-eat-me signaling (CD24–Siglec-10 axis)	Administration not specified; xenograft models	Augmented macrophage phagocytosis; inhibited tumor growth	[126]
	Glycopolymer nanoparticle with MerTK inhibitor (UNC2025)	Inhibit efferocytosis in TAMs to prevent immunosuppression	Mannose-rich polymer for CD206+ TAMs; within TME	Suppressed efferocytosis; prevented anti-inflammatory TAM state	[136]
	ATP-conjugated PLGA nanoparticles	Deliver find-me signaling (ATP) to recruit APCs	Administration not specified; CT26 model	Recruited APCs; synergized with anti-PD-1 for tumor regression	[116]
